# Cuproptosis-related lncRNA signature for prognostic prediction in patients with acute myeloid leukemia

**DOI:** 10.1186/s12859-023-05148-9

**Published:** 2023-02-03

**Authors:** Yidong Zhu, Jun He, Zihua Li, Wenzhong Yang

**Affiliations:** 1 Department of Traditional Chinese Medicine, Shanghai Tenth People’s Hospital, Tongji University School of Medicine, Shanghai, 200072 China; 2 Department of Hematology, Shanghai Punan Hosptial of Pudong New District, Shanghai, 200125 China; 3 Department of Hematology, Shanghai Tenth People’s Hospital, Tongji University School of Medicine, Shanghai, 200072 China; 4 Department of Orthopedics, Shanghai Tenth People’s Hospital, Tongji University School of Medicine, Shanghai, 200072 China

**Keywords:** Cuproptosis, lncRNA signature, Acute myeloid leukemia, Prognostic prediction, Tumor immunity

## Abstract

**Background:**

Long non-coding RNAs (lncRNAs) have been reported to have a crucial impact on the pathogenesis of acute myeloid leukemia (AML). Cuproptosis, a copper-triggered modality of mitochondrial cell death, might serve as a promising therapeutic target for cancer treatment and clinical outcome prediction. Nevertheless, the role of cuproptosis-related lncRNAs in AML is not fully understood.

**Methods:**

The RNA sequencing data and demographic characteristics of AML patients were downloaded from The Cancer Genome Atlas database. Pearson correlation analysis, the least absolute shrinkage and selection operator algorithm, and univariable and multivariable Cox regression analyses were applied to identify the cuproptosis-related lncRNA signature and determine its feasibility for AML prognosis prediction. The performance of the proposed signature was evaluated via Kaplan–Meier survival analysis, receiver operating characteristic curves, and principal component analysis. Functional analysis was implemented to uncover the potential prognostic mechanisms. Additionally, quantitative real-time PCR (qRT-PCR) was employed to validate the expression of the prognostic lncRNAs in AML samples.

**Results:**

A signature consisting of seven cuproptosis-related lncRNAs (namely NFE4, LINC00989, LINC02062, AC006460.2, AL353796.1, PSMB8-AS1, and AC000120.1) was proposed. Multivariable cox regression analysis revealed that the proposed signature was an independent prognostic factor for AML. Notably, the nomogram based on this signature showed excellent accuracy in predicting the 1-, 3-, and 5-year survival (area under curve = 0.846, 0.801, and 0.895, respectively). Functional analysis results suggested the existence of a significant association between the prognostic signature and immune-related pathways. The expression pattern of the lncRNAs was validated in AML samples.

**Conclusion:**

Collectively, we constructed a prediction model based on seven cuproptosis-related lncRNAs for AML prognosis. The obtained risk score may reveal the immunotherapy response in patients with this disease.

**Supplementary Information:**

The online version contains supplementary material available at 10.1186/s12859-023-05148-9.

## Introduction

Acute myeloid leukemia (AML) is a malignant clonal disease of the hematopoietic system, which is characterized by the accumulation of abnormal primitive cells and impaired production of normal blood cells [1]. In 2022, 20,050 newly diagnosed cases and 11,540 deaths of patients with this disease were estimated to occur in the United States [2]. Although induction therapy has achieved complete remission in most patients [3], relapses are still common. In previous publications, a five-year overall survival (OS) of 35–40% was reported in AML patients under 60 years of age, whereas it was only 10–15% for older patients [4, 5]. It is noteworthy that the combination of targeted therapy and chemotherapy may bring breakthrough in the treatment of AML, but such combined treatments are still under exploration and optimization [6]. Therefore, novel prognostic biomarkers are highly needed for the improvement of AML prognosis and treatment.

Copper is among the trace elements essential to the human body, but it can also be harmful if its level reaches beyond a certain threshold concentration [7]. Recently, a copper-triggered modality of mitochondrial cell death was reported, which was termed “cuproptosis” [8]. The functional role of cuproptosis in cancer development has been previously reported. For example, cuproptosis was used to predict clinical outcomes and immune response in bladder cancer [9], breast cancer [10], colorectal cancer [11], and prostate cancer [12]. A more recent study suggested that cuproptosis-related genes might be critically involved in the 2-year AML prognosis and the immune response to treatment [13]. However, the role of cuproptosis in the pathogenesis and the long-term prognosis of AML has not yet been fully elucidated.

Accumulating evidence has suggested that long non-coding RNAs (lncRNAs), a type of non-coding transcripts, exert a functional role at almost all stages of gene expression and are involved in the development of different solid cancer types [15, 16]. In addition to its participation in the pathogenesis of solid cancers, lncRNAs also play critical roles in the development of leukemia, including AML. For instance, the downregulation of lncRNA DLEU7-AS1 was recently found to be a favorable prognostic factor for AML [17]. Additionally, lncRNA-LOC100506453 was indicated as a noninvasive biomarker for acute promyelocytic leukemia (APL, a subtype for AML) treatment surveillance [18]. Furthermore, in a previous study, lncRNA H22954 inhibited AML angiogenesis [19]. In recent years, increasingly more studies have suggested that lncRNAs are potential biomarkers for the prognosis of complex diseases and clinical outcome prediction [16]. The application of lncRNA signatures for AML prognosis prediction has also been reported. Several recent studies confirmed that N6-methyadenosine-related lncRNAs predicted AML prognosis and the immune landscape [20–22]. Moreover, it is worth mentioning that cuproptosis-related lncRNA signature also showed promising prognosis prediction potential in carcinomas, including hepatocellular carcinoma [23], lung adenocarcinoma [24], colon adenocarcinoma [25], and colorectal cancer [26]. However, whether cuproptosis-related lncRNA signature could be utilized for the prediction of AML prognosis and immune response is largely unknown.

This study aimed to identify cuproptosis-related lncRNAs associated with AML prognosis and to evaluate their prognosis prediction value. Based on the identified lncRNAs, a prediction model was constructed and the expression of the lncRNAs was validated by quantitative real-time PCR (qRT-PCR) in clinical AML samples.

## Materials and methods

### Data collection

The RNA sequencing (RNA-seq) and clinical characteristics data of AML patients were obtained from The Cancer Genome Atlas (TCGA) database [27]. Patients with incomplete data and pediatric AML were excluded from the subsequent analysis. Based on literature review [7, 14, 28–31], a total number of 19 cuproptosis-related genes were subjected to analysis (Additional file 1: Table S1).

### Correlation evaluation

The GENCODE annotation file [32] was used to identify the obtained lncRNAs. The co-expression relationship between cuproptosis-related genes and lncRNAs in AML samples was established via Pearson correlation analysis. Correlation coefficients (|Pearson R|) > 0.6 and *P-*values < 0.001 were employed as criteria to indicate lncRNAs that were closely related to cuproptosis.

### Construction and validation of the prognostic model

The cases were randomized at a ratio of 1:1 into a training cohort and a validation cohort. Cuproptosis-related lncRNAs that correlated with the prognosis of AML were screened using univariable Cox regression analysis at *P-*value < 0.05. The least absolute shrinkage and selection operator (LASSO) regression was adopted to identify the optimal panel of prognostic lncRNAs. Then, multivariable Cox regression analysis was applied to develop a risk model based on the obtained lncRNAs extracted by the LASSO method. The survival risk score was next calculated using the following formula:

$${\text{Risk Score }} = \, \sum {\left[ {{\text{Exp }}\left( {{\text{lncRNA}}} \right) \times {\text{coef }}\left( {{\text{lncRNA}}} \right)} \right]} \,$$

Further, based on their median risk scores, the samples were separated into two groups: a high- and a low-risk group. Kaplan–Meier curves were implemented to evaluate the survival discrepancy, and the obtained data were analyzed statistically by the log-rank test. Univariable and multivariable Cox regression analyses were performed to evaluate the value of the clinical characteristics and risk score for prognosis prediction. Receiver operating characteristic (ROC) and C-index curves were constructed to investigate the accuracy and specificity of the proposed model. Stratified survival analysis based on the clinical characteristics was conducted to evaluate the applicability of the cuproptosis-related signature. These measurements were analyzed and visualized by “survival”, “caret”, “limma”, “glmnet”, “survminer”, “timeROC”, “pheatmap”, “rms”, and “pec” packages in R software (version 4.1.3; http://www.r-project.org).

### Principal component analysis (PCA) and nomogram development

PCA was utilized to visualize the high-dimensional data of the whole genes, cuproptosis-related genes, cuproptosis-related lncRNAs, and the lncRNAs involved in the risk model construction. Then, to forecast the 1-, 3-, and 5-year OS, a nomogram was developed, which consisted of the risk score and the clinical features. The calibration curve was utilized to examine the predictive ability of the established model. These analyses were performed and visualized by “limma”, “scatterplot3d”, “survival”, “regplot”, and “rms” packages.

### Functional enrichment analysis

To investigate the possible mechanisms, Gene Ontology (GO) and Kyoto Encyclopedia of Genes and Genome (KEGG) analyses were performed based on the identified differentially expressed genes. In addition, the gene set enrichment analysis (GSEA) software [33] was utilized to identify the significantly enriched pathways between the low- and high-risk groups. The single sample gene set enrichment analysis (ssGSEA) score was used to discriminate the enrichment levels of immune-related functions and cells between the high- and low-risk groups. The data were evaluated and visualized by “limma”, “clusterProfiler”, “enrichplot”, “DOSE”, “pheatmap”, “GSVA”, “GSEABase”, and “reshape2” packages.

### qRT-PCR

Peripheral blood samples from 10 AML patients and 10 healthy volunteers were collected, and mononuclear cells were isolated to validate the expression pattern of the identified prognostic lncRNAs. The samples of the AML patients should meet the criterion of a percentage of peripheral blood archaeocytes > 20%. Total RNA was extracted from mononuclear cells using the Trizol (Takara, Japan) and was then reverse transcribed to cDNA using the RevertAid First Strand cDNA Synthesis Kit (Thermo Scientific, USA). PCR was performed using SsoFastTM EvaGreen Supermix (Bio-Rad, USA) according to the manufacturer’s instructions. The gene expression was normalized to GAPDH using the 2^−ΔΔCt^ method. The primers utilized for qRT-PCR are presented in Table 1. This study was approved by the Ethics Committee of Shanghai Tenth People's Hospital (22K159). Informed consent was obtained from all subjects.

**Table 1:** Primers used for qRT-PCR

Gene name	Strand	5’–3’
*NFE4*	Forward	TTGGGGAATGGATGCCACAA
	Reverse	GCCGCACACAGTTGCTTAAA
*LINC00989*	Forward	GAGTTTTCAGTGGCAAGCCG
	Reverse	GACAGGATTTAGCGCTGGGA
*LINC02062*	Forward	GAGGCTGTCGGACTCTGACT
	Reverse	GATGCTCTGGGATGCTGGTA
*AC006460.2*	Forward	CCCAAAGGAGAGCAGTGAGG
	Reverse	GCTCTAGCCTGCTGGAAGAG
*AL353796.1*	Forward	ACTCATACTCCAAGCACGGC
	Reverse	TTTTTGCACACCCACACAGC
*PSMB8-AS1*	Forward	CCTCTAAACCCCGCCTCTTC
	Reverse	AGTGCTTCTCATCACCCAGC
*AC000120.1*	Forward	ATGGAGGTTTCAGCCATGCA
	Reverse	ACACCTGATGTCCTGGAGGA

### Statistical analysis

Data analysis was performed using R software (version 4.1.3) and GraphPad Prism (version 8.0.1, GraphPad Software, San Diego, CA, USA). Wilcoxon test was performed to compare two independent groups, and chi-square analysis was conducted to assess categorical variables. Two-sided *P* < 0.05 was considered to indicate statistically significant differences.

## Results

### Identification of cuproptosis-related lncRNAs

**Fig. 1:**
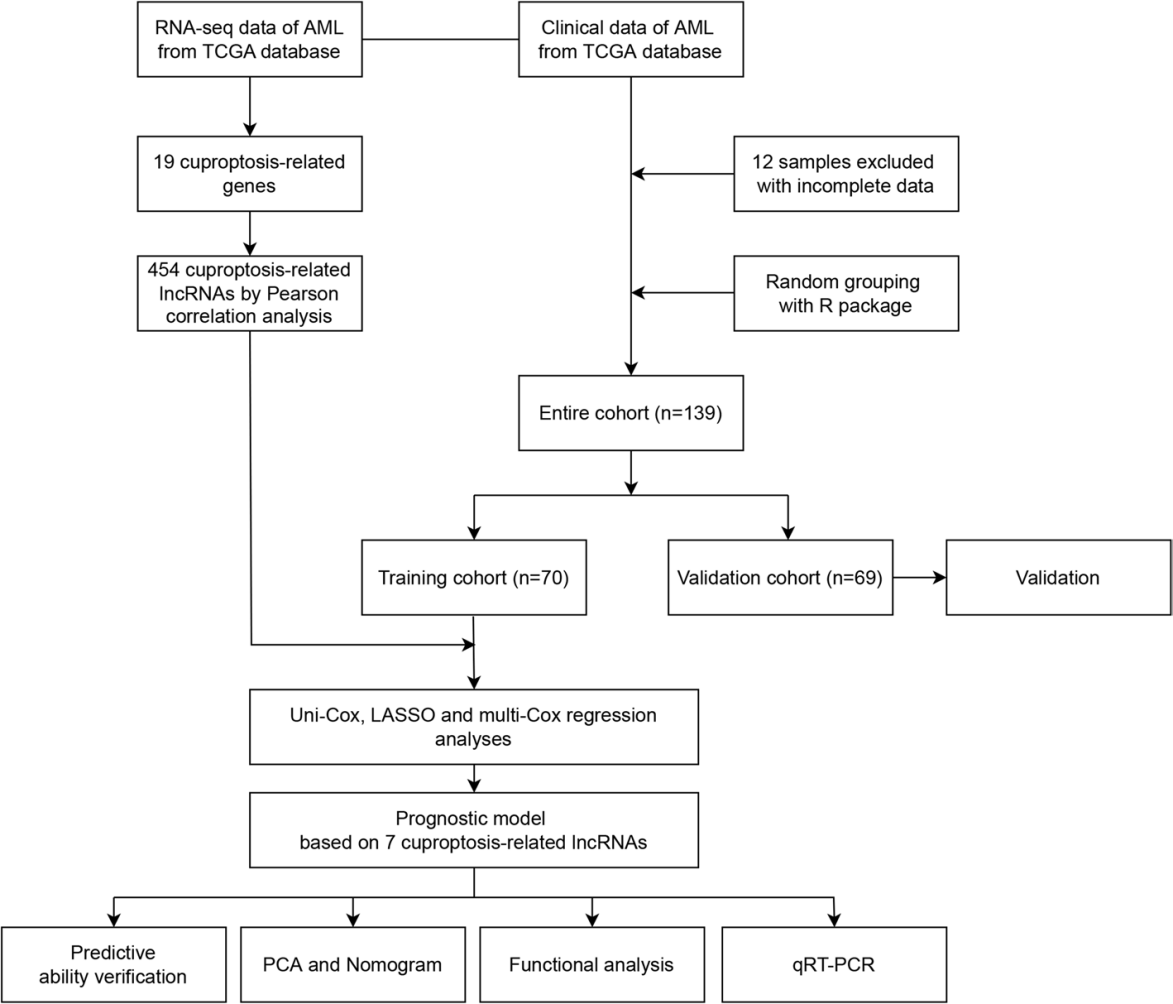
Study flowchart

**Fig. 2:**
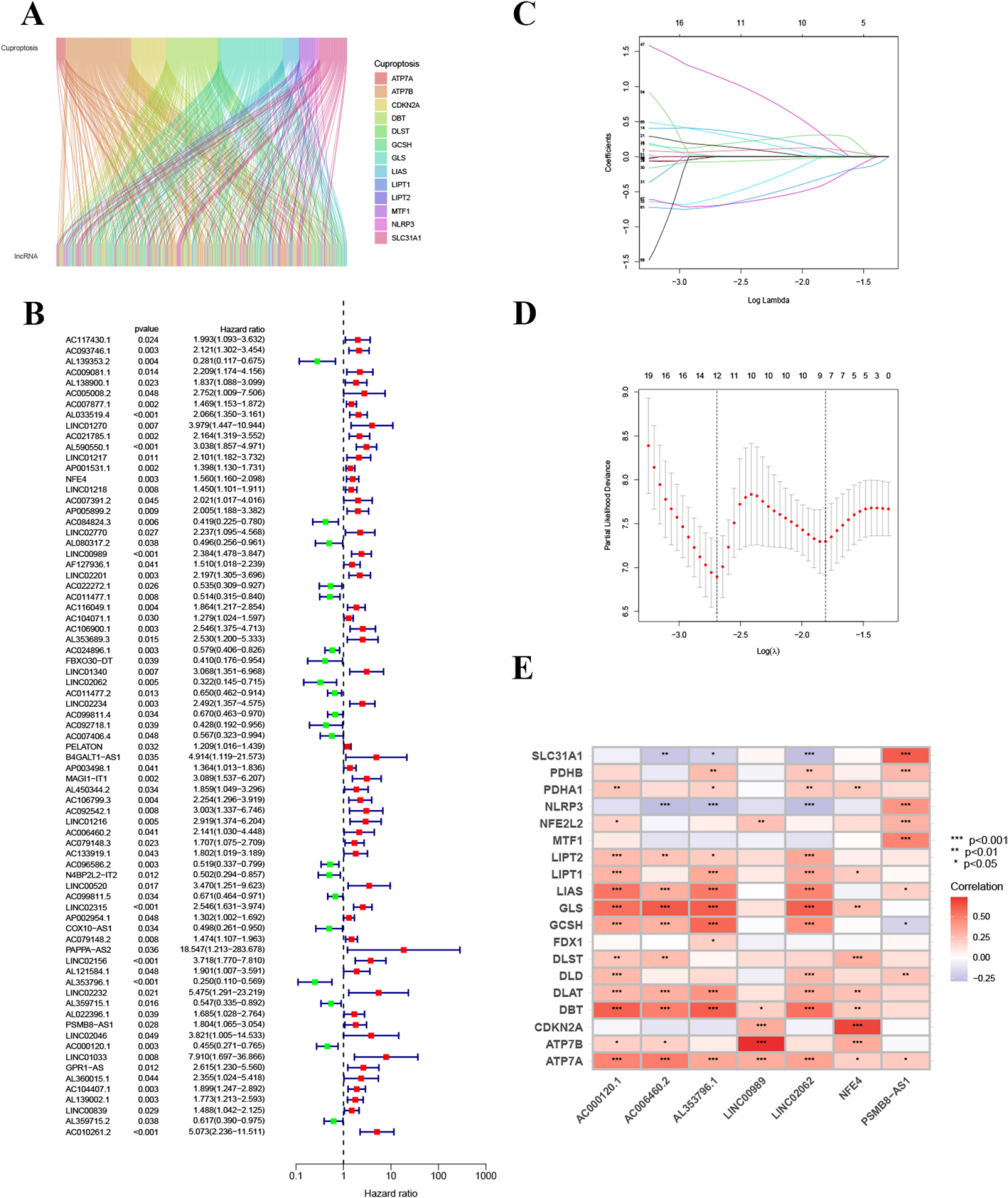
Identification of cuproptosis-related lncRNA signatures. **A** Sankey diagram of the correlation between the cuproptosis-related genes and lncRNAs; **B** The forest map of the cuproptosis-related lncRNAs correlated with OS; **C**, **D** The LASSO analysis of the prognosis-linked lncRNAs; **E** Correlational heatmap for the lncRNAs involved in the model construction and cuproptosis-related genes

The flowchart including the identification of the lncRNA signature, the nomogram construction, and the subsequent analyses is displayed in Fig. 1. The RNA-seq data of 151 AML samples were obtained from the TCGA database, and 12 samples with incomplete data were excluded. Finally, 139 samples with unabridged clinical data were subjected to analysis. Based on the GENCODE annotation file, 16,876 lncRNAs were identified. Using the identified 19 cuproptosis-related genes, a total number of 454 cuproptosis-related lncRNAs with a co-expression relationship in AML were identified (Fig. 2A, Additional file 2: Table S2).

### Construction and validation of the prognostic model

The patients were randomly divided into a training cohort (*n* = 70) and a validation cohort (*n* = 69). No statistical difference in clinical features was established between the two cohorts (Table 2). A total number of 75 cuproptosis-related lncRNAs were significantly correlated with OS of AML patients, which were identified by univariable Cox regression analysis (Fig. 2B). Next, LASSO analysis was implemented to extract potential cuproptosis-related lncRNA signatures for prognostic prediction in AML patients, resulting in the identification of 12 lncRNAs (Fig. 2C, D). Afterwards, multivariable Cox regression analysis revealed that seven cuproptosis-related lncRNAs (NFE4, LINC00989, LINC02062, AC006460.2, AL353796.1, PSMB8-AS1, and AC000120.1) were independent risk factors for AML prognosis (Fig. 2E, Additional file 3: Table S3). The risk score was calculated using the following formula:

$$\begin{aligned} {\text{Risk Score }} = \, & \left( {0.{6578 } \times {\text{ NFE4 expression}}} \right) \, + \, \left( {0.{4613 } \times {\text{ LINC}}00{\text{989 expression}}} \right) \, \\ + \, \left( { - {1}.{2572 } \times {\text{ LINC}}0{2}0{\text{62 expression}}} \right) \, + \, \left( {{2}.00{24 } \times {\text{ AC}}00{646}0.{\text{2 expression}}} \right) \, \\ + \, \left( { - {1}.{264}0 \, \times {\text{ AL353796}}.{\text{1 expression}}} \right) + \, \left( {0.{7749 } \times {\text{ PSMB8}} - {\text{AS1 expression}}} \right) \\ + \, \left( { - 0.{9596 } \times {\text{ AC}}000{12}0.{\text{1 expression}}} \right). \\ \end{aligned}$$

**Table 2:** Demographical characteristics

Variables		Whole cohort	Training cohort	Validation cohort	*P-*value
Age	≤ 65	99 (71.22%)	49 (70%)	50 (72.46%)	0.89
	> 65	40 (28.78%)	21 (30%)	19 (27.54%)	
Gender	Female	62 (44.60%)	30 (42.86%)	32 (46.38%)	0.81
	Male	77 (55.40%)	40 (57.14%)	37 (53.62%)	

**Fig. 3:**
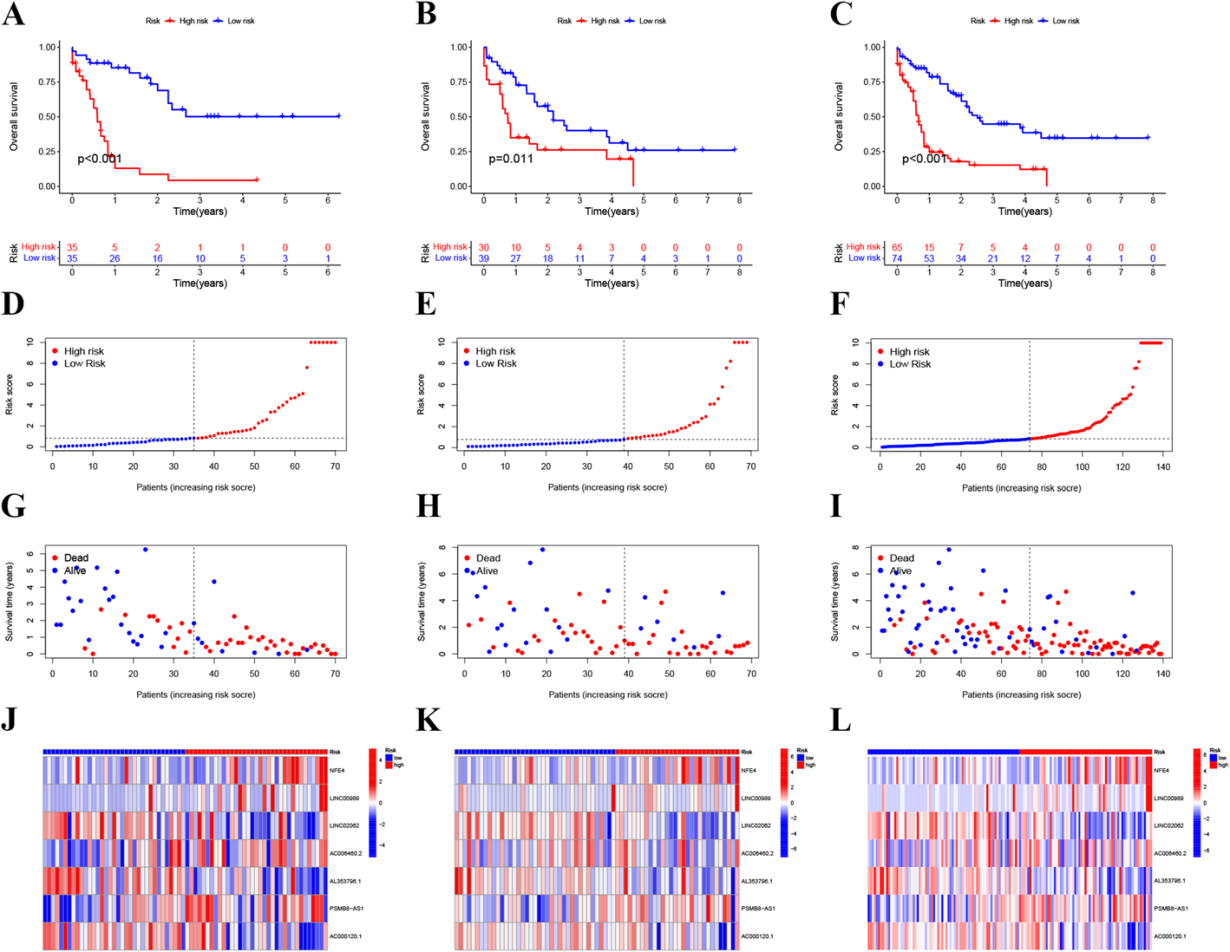
Prognosis based on the seven cuproptosis-related lncRNA signatures. **A**–**C** Kaplan–Meier survival curves of overall survival of AML samples in the training (**A**), validation (**B**), and whole cohorts (**C**); **D**–**F** Risk scores of the AML samples in the training (**D**), validation (**E**), and whole cohorts (**F**). **G**–**I** Survival status of the AML samples in the training (**G**), validation (**H**), and whole cohorts (**I**); **J**–**L** Heatmaps of the expression levels of the seven cuproptosis-related lncRNAs in the training (**J**), validation (**K**), and whole cohorts (**L**)

The Kaplan–Meier curves revealed that the OS ratio of the AML samples in the low-risk group was significantly higher than that in the high-risk group in the training, validation, and whole cohorts (Fig. 3A–C). The risk plots indicated that an increased number of death cases was associated with the higher risk score in the training, validation, and whole cohorts (Fig. 3D–I). The heatmaps illustrated the expression of the seven cuproptosis-related lncRNA signatures in the high- and low-risk groups (Fig. 3J–L). Among them, LINC00989, NFE4, PSMB8-AS1, and AC006460.2 were significantly upregulated, whereas AL353796.1, LINC02062, and AC000120.1 were significantly downregulated in the high-risk group.

**Fig. 4:**
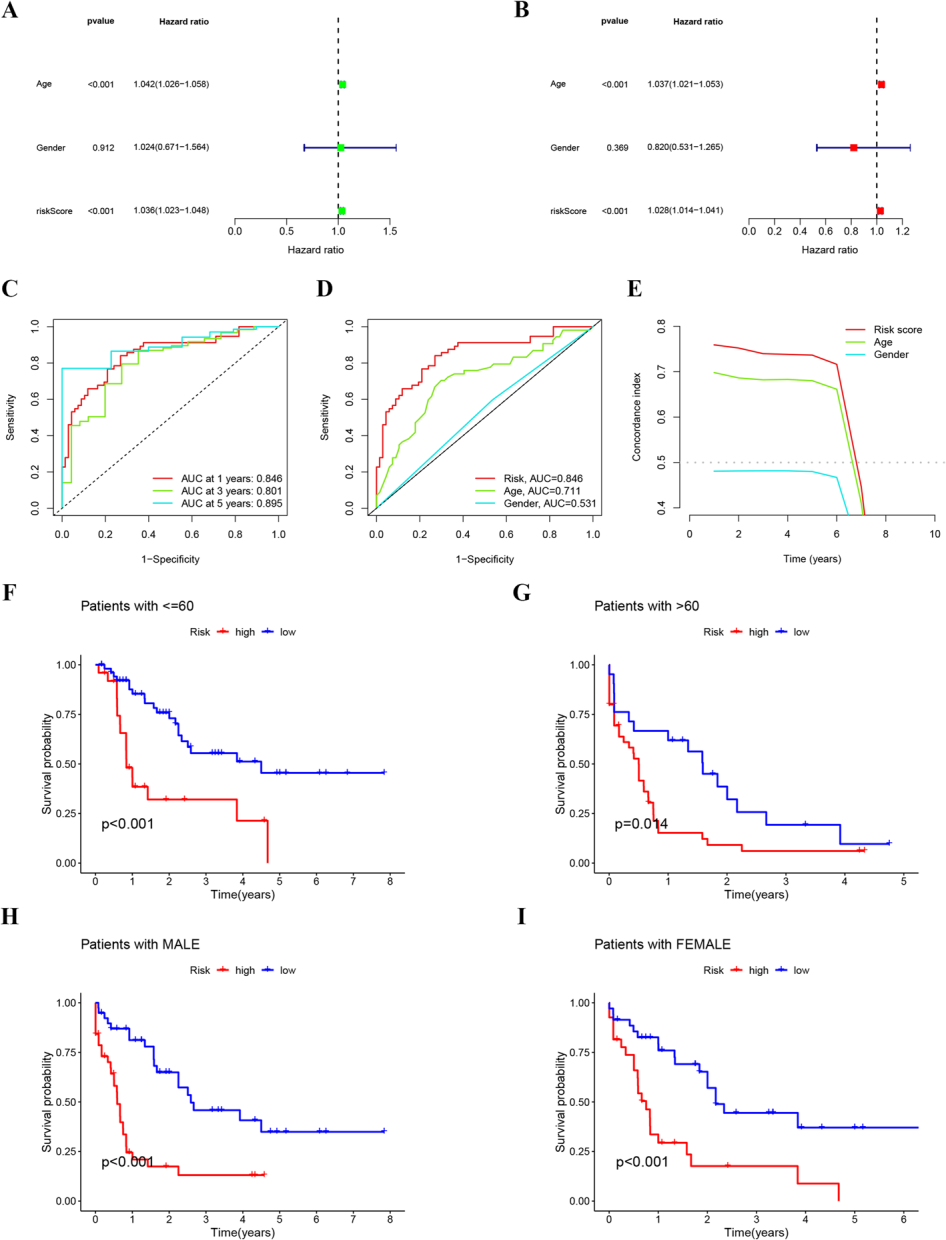
Further verification of the risk model. **A**, **B** Univariable Cox (**A**) and multivariable Cox regression analysis (**B**); **C** The ROC curves of the risk model at 1, 3, and 5 years; **D** The ROC curves of the clinical features and risk score; **E** The C-index curves of the clinical features and risk score; **F**–**I** Kaplan–Meier survival curves of the risk model classified by age and gender, respectively

The univariable and multivariable Cox regression analyses showed that both age and risk score were significantly associated with the OS of AML patients and served as independent prognostic factors (Fig. 4A, B). The AUC values of the 1-, 3-, and 5-year survival were 0.846, 0.801, and 0.895, respectively (Fig. 4C). Furthermore, the constructed ROC curves (Fig. 4D) and C-index curves (Fig. 4E) revealed that the risk score performed better in predicting the AML prognosis than the other clinical features. Then, the patients were stratified by age and gender to evaluate the application of the risk score in prognosis prediction. The low-risk group had significantly higher OS than the high-risk group regardless of age (≤ 60 or > 60) (Fig. 4F–G) or gender (Fig. 4H–I). These findings indicated that the signature composed of seven cuproptosis-related lncRNAs successfully predicted the prognostic risk of AML.

### PCA and nomogram development

**Fig. 5:**
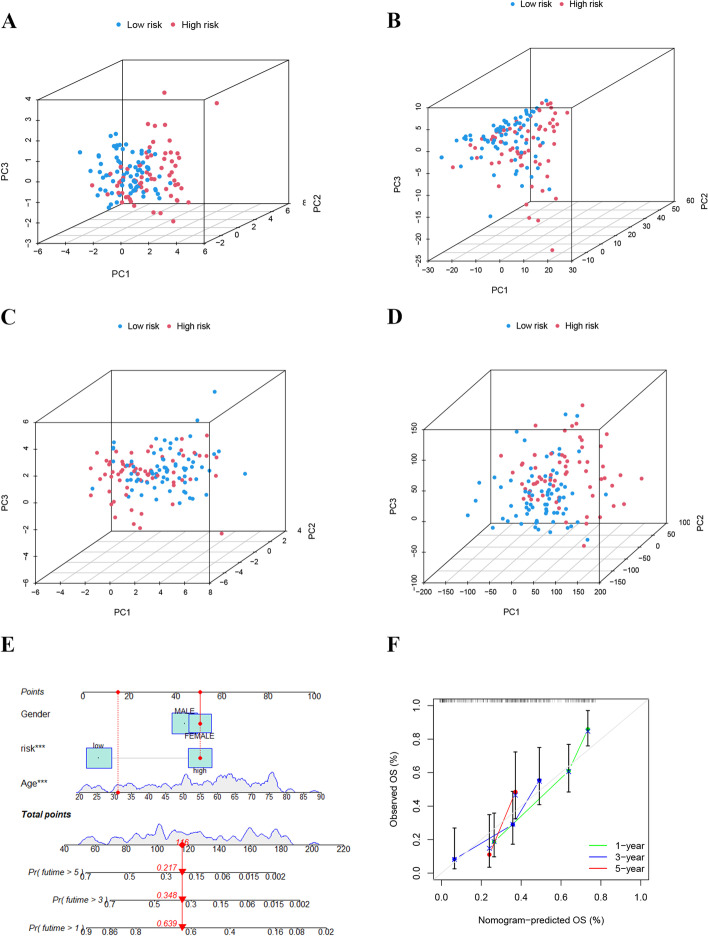
PCA and nomogram development. **A**–**D** PCA distributed by model-constructed lncRNAs (**A**), cuproptosis‐related lncRNAs (**B**), cuproptosis‐related genes (**C**), and all genes (**D**); **E** The nomogram used for the prediction of the 1-, 3-, and 5-year overall survival; (**F**) Calibration curves

PCA was employed to visualize the distribution of the model-constructed lncRNAs (Fig. 5A), cuproptosis‐related lncRNAs (Fig. 5B), cuproptosis‐related genes (Fig. 5C), and all genes (Fig. 5D). Among them, the lncRNAs involved in the risk model construction had the most obvious distribution. Next, a nomogram based on the risk score and clinical features was created to predict the 1-, 3-, and 5-year OS (Fig. 5E). The calibration curves indicated the optimal concordance between the practical observation and the predicted survival rates (Fig. 5F). These results suggest a good performance of the nomogram model in AML prognosis prediction.

### Functional enrichment analysis

**Fig. 6:**
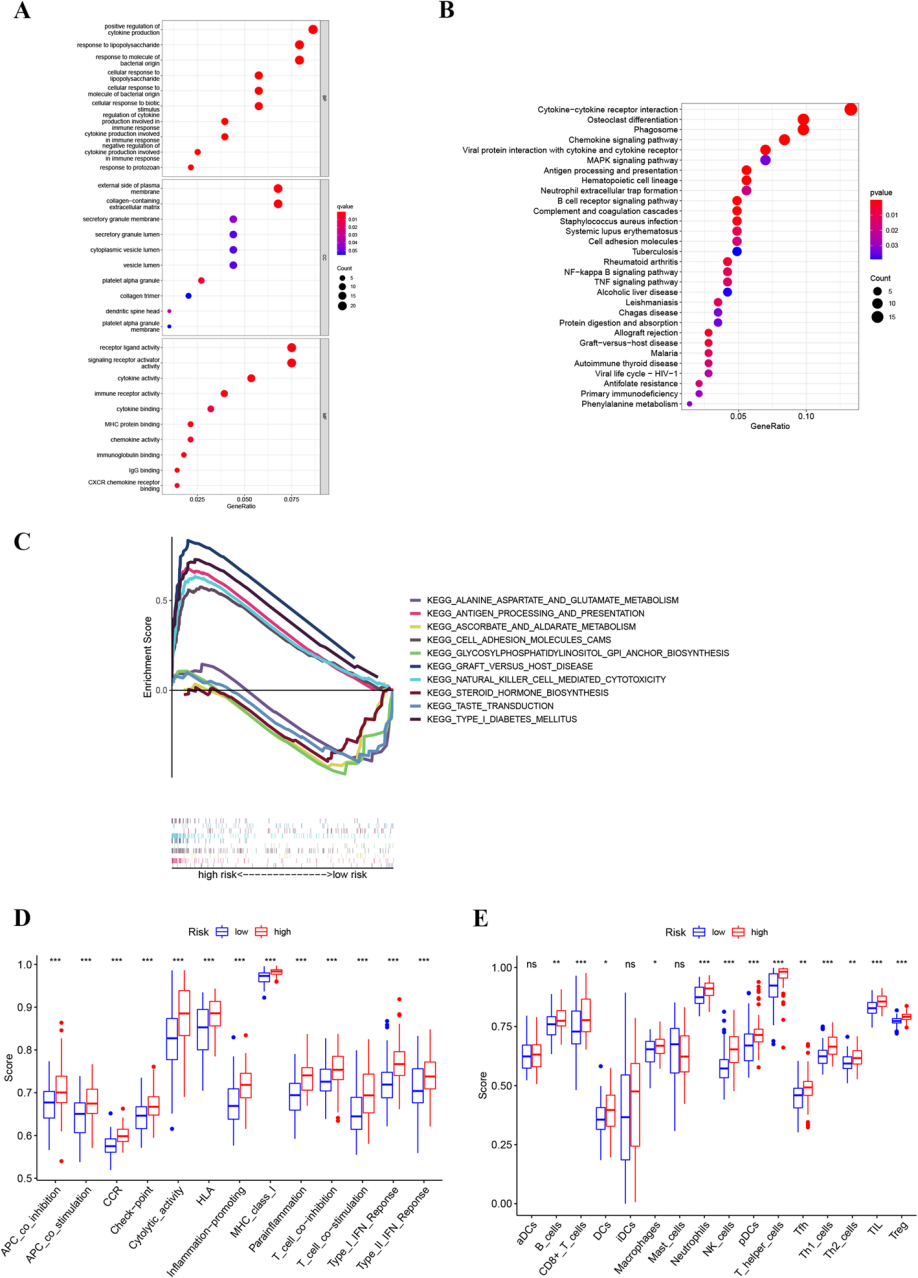
Functional enrichment analysis. **A** The bubble diagram of the Gene Ontology (GO) analysis; **B** The bubble diagram of the Kyoto Encyclopedia of Genes and Genome (KEGG) analysis; **C** Enrichment plot of GSEA; **D** The boxplot of the immune-related functions; **E** The boxplot of the immune-related cells

Functional enrichment analysis was conducted to explore the possible mechanisms by which lncRNA signatures were involved in prognosis prediction. The differential genes between the high- and low-risk groups were identified and subjected to further analysis (Additional file 4: Table S4). GO function analysis indicated that the biological processes included mainly positive regulation of cytokine production, response to lipopolysaccharides, and response to molecules of bacterial origin (Fig. 6A). The KEGG analysis results revealed predominantly cytokine-cytokine receptor interactions, osteoclast differentiation, and phagosome formation (Fig. 6B). GSEA showed that the pathways enriched in the high-risk group were highly correlated with immunity, such as natural killer cell-mediated cytotoxicity and antigen processing and presentation, whereas metabolism-related pathways, for instance, alanine, aspartate, and glutamate metabolism, as well as ascorbate and aldarate metabolism, were critical pathways in the low-risk group (Fig. 6C). Furthermore, ssGSEA analysis disclosed that all immune-related functions and 13 of 16 immune-related cells differed significantly between the high- and low-risk groups (Fig. 6D, E).

### Validation of the expression of prognostic lncRNAs

**Fig. 7:**
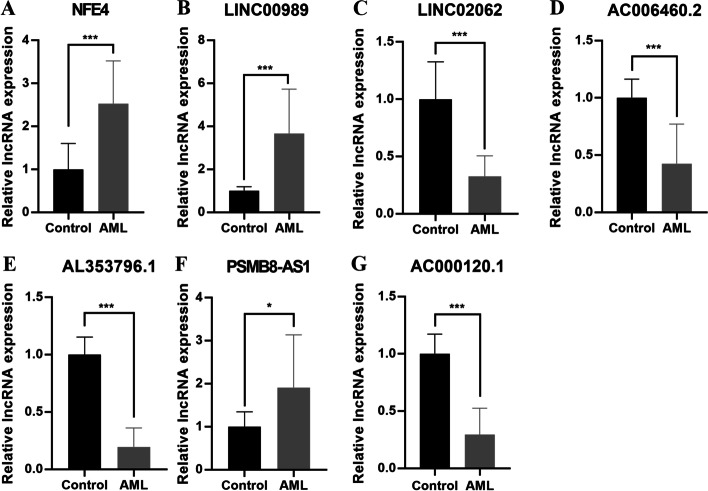
qPCR analysis. **A** NFE4; **B** LINC00989; **C** LINC02062; **D** AC006460.2; **E** AL353796.1; **F** PSMB8-AS1; **G**, AC000120.1. *p < 0.05; ***p < 0.001

Next, qRT-PCR was applied to validate the expression of the prognostic lncRNAs in AML patients and healthy volunteers. Within expectation, compared with healthy volunteers, the expression levels of AC006460.2, AC000120.1, AL353796.1, and LINC02062 were significantly lower in AML patients, whereas those of NFE4, LINC00989 and PSMB8-AS1 were significantly higher (Fig. 7).

## Discussion

The primary finding of the present study is the construction of a prediction model for short- and long-term prognosis in AML patients based on a signature of seven cuproptosis-related lncRNAs. The construction of the prognosis prediction model might facilitate clinical decision making in the implementation of AML treatment and follow-up strategies.

It should be noted that the use of cuproptosis-related lncRNAs for prognostic prediction has been reported in various cancers, including osteosarcoma [34], colon cancer [25], gastric cancer [35], hepatocellular carcinoma [23], and head and neck squamous carcinoma [36]. A recent study identified four cuproptosis-related lncRNAs involved in the prediction of the 2-year OS outcomes in AML patients [13]. In the present study, seven cuproptosis-related lncRNAs were utilized in the construction of a prognostic model for 1-, 3-, and 5-year AML prognosis prediction, which showed good prediction performance (all AUC > 0.8). The constructed model may serve as a powerful tool for AML prognosis prediction. Of the identified lncRNAs, PSMB8-AS1 was previously found to regulate cell proliferation, apoptosis, and radio-resistance in glioblastoma [37]. In another earlier study, the modulation of miR-574-5p/RAB10 expression by PSMB8-AS1 promoted the proliferation of glioma cells [38]. Moreover, the preferential expression of the gamma-globin genes was regulated by NFE4, which was an indispensable component in the prognostic model of clear-cell renal-cell carcinoma [39, 40]. LINC00989 was confirmed to be associated with the OS prognosis of patients with breast cancer and hepatocellular carcinoma [41, 42]. These findings suggest that these lncRNAs play biological roles in the pathogenesis of solid tumors, but their functional role in AML needs further investigation. It is noteworthy that the functional roles of LINC02062, AC006460.2, AL353796.1, and AC000120.1 in the development of complex diseases have not been previously reported. Therefore, our findings provide novel insights into the pathogenesis of AML.

To validate the clinical significance of the proposed signature, the AML patients were divided into a high- and a low-risk group. The low-risk group had a better prognosis than the high-risk group in all cohorts. A previous study established that the long-term survival of AML patients declined with age [5], which is in agreement with the findings of the present investigation. The AUC values of the risk scores for the 1-, 3-, and 5-year prognosis predictions were all above 0.846, indicating a good predicting performance. In addition, the stratified survival analysis revealed that the established model had good performance in different age and gender subgroups, and can thus have a wide clinical application. To further validate the performance of the model, we performed PCA, whose results showed that the lncRNAs in the constructed model had the highest distinction, indicating that they could be used to discriminate among patients from different risk groups. These findings suggested that the model established by the seven cuproptosis-related lncRNAs was reliable for AML prognosis prediction.

To determine the possible mechanism involved in AML survival, several functional enrichment analyses were performed. GSEA demonstrated that the immune-associated pathways were enriched in the high-risk group. Interestingly, the ssGSEA analysis results revealed that all the immune-related functions and 13 of the 16 immune-related cells were significantly enriched. Notably, all immune-related functions and cells were overexpressed in the high-risk group. T-cell co-inhibition was found to be a vital element contributing to immune function suppression by providing inhibitory signals to activated T cells [43, 44]. Furthermore, immune-linked processes, including T-cell co-stimulation and antigen presentation, were significantly correlated with post-transplantation relapses in AML [45]. In the present study, macrophages and Tregs were significantly upregulated in the high-risk group. Tregs are highly immune-suppressive and considered as pivotal regulators of immune escape for inhibiting the proliferation and function of immune killer cells through cellular contact and inhibitory cytokine production [46]. Macrophages that reside within the tumor microenvironment are known as tumor-associated macrophages, which cause immune suppression through enhanced angiogenesis, metastasis, and chemoresistance [47]. The increase in Treg and macrophage levels is associated with a poorer survival in AML [48, 49]. Furthermore, T- and NK-cell exhaustion and dysfunction, which contribute to immune disorder and tumor immune escape [50], were correlated with therapeutic reactivity, high risk for relapse, and unfavorable prognosis of AML [51]. Therefore, we speculated that cuproptosis-related lncRNAs may modulate the tumor microenvironment and promote tumor immune evasion through inhibitory immune cells, resulting in favor of leukemia cell survival. Therefore, it could be reasonable to infer that a possible tight connection might also exist between cuproptosis and tumor immunity in AML.

Beyond lncRNAs, it should be noted that microRNAs (miRNAs) and circular RNAs (circRNAs) have been used for clinical outcomes prediction and disease treatment in complex diseases [52–54]. The prediction model in this study was constructed using lncRNAs, but miRNAs or circRNAs were not utilized. Although the 5-year prediction performance reached a value of 0.895, model prediction ability improvement is still needed. A mixed model containing miRNAs, lncRNAs, circRNAs, and other non-coding RNAs may be included in a future computational model for complex diseases prognosis and immune response prediction. In addition, in the present investigation, we used conventional strategies for model development, and some lncRNAs that have impact on prognosis might have been excluded from our analysis. Therefore, machine learning-based or other models of computational strategies (such as a network algorithm-based approach) may be recommended for comparative assessments.

In the present study, seven cuproptosis-related lncRNAs were identified. Of them, four lncRNAs have not been previously reported in the literature. Therefore, lncRNA research in AML has been insufficient, and our findings provide novel insights into the pathogenesis of this disease. Moreover, it could be observed that the AUC value for 5-year prediction was near 0.9, which suggested a robust and high potential of the prognosis prediction model and the possibility for its effective implementation in clinical practice. Moreover, the newly developed model performed well in all age subgroups, which indicated a wide clinical application. Nevertheless, this study is not without limitations. The performance of this model was confirmed by a validation cohort derived from only one database, which needed an external validation. Although the differential expression of the prognostic lncRNAs was validated by qRT-PCR, more prospective investigations are needed to confirm its predictive performance.

## Conclusion

In conclusion, the model based on the seven newly identified cuproptosis-related lncRNAs has a good prognostic value for clinical outcomes in AML patients. Immune-related pathways might be involved in the lncRNA signature-associated survival. However, future research is needed to confirm the performance of this prediction model and the potential for its application in clinical practice.

## Supplementary Information

**Additional file 1. Table S1** Cuproptosis-related genes.

**Additional file 2. Table S2** Co-expression relationship between cuproptosis-related genes and lncRNAs.

**Additional file 3. Table S3** Regression coefficients of cuproptosis-related lncRNAs determined via multi-Cox analysis.

**Additional file 4. Table S4** Differential genes between the high- and low-risk groups.

**Additional file 5.** Data analysis results.

## Data Availability

Access to public data are available in The Cancer Genome Atlas (TCGA) (https://portal.gdc.cancer.gov/repository?facetTab=files&filters=%7B%22op%22%3A%22and%22%2C%22content%22%3A%5B%7B%22op%22%3A%22in%22%2C%22content%22%3A%7B%22field%22%3A%22cases.project.program.name%22%2C%22value%22%3A%5B%22TCGA%22%5D%7D%7D%2C%7B%22op%22%3A%22in%22%2C%22content%22%3A%7B%22field%22%3A%22cases.project.project_id%22%2C%22value%22%3A%5B%22TCGA-LAML%22%5D%7D%7D%2C%7B%22op%22%3A%22in%22%2C%22content%22%3A%7B%22field%22%3A%22files.analysis.workflow_type%22%2C%22value%22%3A%5B%22STAR%20-%20Counts%22%5D%7D%7D%2C%7B%22op%22%3A%22in%22%2C%22content%22%3A%7B%22field%22%3A%22files.data_category%22%2C%22value%22%3A%5B%22transcriptome%20profiling%22%5D%7D%7D%2C%7B%22op%22%3A%22in%22%2C%22content%22%3A%7B%22field%22%3A%22files.data_format%22%2C%22value%22%3A%5B%22tsv%22%5D%7D%7D%2C%7B%22op%22%3A%22in%22%2C%22content%22%3A%7B%22field%22%3A%22files.data_type%22%2C%22value%22%3A%5B%22Gene%20Expression%20Quantification%22%5D%7D%7D%5D%7D) database. The related data analysis was provided in the Additional file 5. Experimental data of our study is available from the corresponding author on reasonable request.
