# Supplementary material for: Cuproptosis-related lncRNA signature for prognostic prediction in patients with acute myeloid leukemia
Source: BMC Bioinformatics. 2023 Feb 3;24:37. doi: 10.1186/s12859-023-05148-9 (PMC9896718; doi:10.1186/s12859-023-05148-9)
Supplement: Supplementary file 1 — Additional file 1. Table S1 Cuproptosis-related genes. [file 12859_2023_5148_MOESM1_ESM.docx]

| **Cuproptosis-related Genes** |
| --- |
| NFE2L2 |
| NLRP3 |
| ATP7B |
| ATP7A |
| SLC31A1 |
| FDX1 |
| LIAS |
| LIPT1 |
| LIPT2 |
| DLD |
| DLAT |
| PDHA1 |
| PDHB |
| MTF1 |
| GLS |
| CDKN2A |
| DBT |
| GCSH |
| DLST |

**Supplementary Table S1. Cuproptosis-related Genes**
