# Supplementary material for: Cuproptosis-related lncRNA signature for prognostic prediction in patients with acute myeloid leukemia
Source: BMC Bioinformatics. 2023 Feb 3;24:37. doi: 10.1186/s12859-023-05148-9 (PMC9896718; doi:10.1186/s12859-023-05148-9)
Supplement: Supplementary file 2 — Additional file 2. Table S2 Co-expression relationship between cuproptosis-related genes and lncRNAs. [file 12859_2023_5148_MOESM2_ESM.docx]

**Supplementary Table S2. Co-expression relationship between cuproptosis-related genes and lncRNAs**

| **Cuproptosis-related genes** | **lncRNA** | **cor** | **p** |
| --- | --- | --- | --- |
| CDKN2A | AC055874.1 | 0.74519959 | 7.89E-28 |
| ATP7A | AL731566.2 | 0.617509164 | 3.89E-17 |
| ATP7B | GRM3-AS1 | 0.656008407 | 8.14E-20 |
| DBT | AP000813.1 | 0.600092258 | 4.84E-16 |
| ATP7B | AC117430.1 | 0.76668454 | 2.80E-30 |
| DBT | AC108449.2 | 0.660582423 | 3.68E-20 |
| CDKN2A | AC137770.1 | 0.615284551 | 5.41E-17 |
| GLS | AL078600.1 | 0.607585917 | 1.67E-16 |
| GLS | LAMTOR5-AS1 | 0.619193698 | 3.02E-17 |
| SLC31A1 | AL139041.1 | 0.640432099 | 1.10E-18 |
| CDKN2A | AC093746.1 | 0.791966103 | 1.60E-33 |
| LIAS | AC012063.1 | 0.687395409 | 2.63E-22 |
| LIAS | PAXIP1-AS2 | 0.739681495 | 3.07E-27 |
| SLC31A1 | AC105265.3 | 0.619114107 | 3.06E-17 |
| ATP7B | MRPS30-DT | 0.712639065 | 1.50E-24 |
| SLC31A1 | AL445524.1 | 0.65923931 | 4.65E-20 |
| MTF1 | AL445524.1 | 0.62077168 | 2.38E-17 |
| GLS | AL162595.1 | 0.613455419 | 7.09E-17 |
| ATP7B | LINC00570 | 0.749752759 | 2.50E-28 |
| ATP7B | AL137024.1 | 0.716293013 | 6.77E-25 |
| LIAS | AL139353.2 | 0.71298843 | 1.39E-24 |
| GLS | AL139353.2 | 0.632498192 | 3.90E-18 |
| DBT | AL139353.2 | 0.679082218 | 1.29E-21 |
| GLS | AL592295.6 | 0.646533614 | 4.03E-19 |
| DBT | AL592295.6 | 0.679013875 | 1.30E-21 |
| GLS | AC012360.3 | 0.628232006 | 7.60E-18 |
| CDKN2A | AC009081.1 | 0.788460961 | 4.79E-33 |
| SLC31A1 | AC109597.2 | 0.602165706 | 3.61E-16 |
| MTF1 | AC109597.2 | 0.626687611 | 9.65E-18 |
| NLRP3 | AC098869.2 | 0.620850857 | 2.35E-17 |
| SLC31A1 | AC098869.2 | 0.778552793 | 9.50E-32 |
| MTF1 | AC098869.2 | 0.719323717 | 3.47E-25 |
| GLS | TMEM161B-AS1 | 0.658584685 | 5.22E-20 |
| DBT | TMEM161B-AS1 | 0.60591753 | 2.12E-16 |
| CDKN2A | AL449983.1 | 0.716575612 | 6.36E-25 |
| CDKN2A | AL138900.1 | 0.784691642 | 1.52E-32 |
| ATP7B | AC005008.2 | 0.752272684 | 1.31E-28 |
| ATP7B | AL451047.1 | 0.711848377 | 1.78E-24 |
| SLC31A1 | AC007877.1 | 0.658597572 | 5.20E-20 |
| LIAS | AC022306.2 | 0.602789607 | 3.31E-16 |
| GLS | AC022306.2 | 0.669833703 | 7.09E-21 |
| DBT | AC022306.2 | 0.653266167 | 1.30E-19 |
| ATP7B | AC127526.4 | 0.74659729 | 5.56E-28 |
| CDKN2A | AC127526.4 | 0.606055703 | 2.08E-16 |
| GLS | LINC01534 | 0.653982442 | 1.15E-19 |
| LIAS | LINC00909 | 0.617796405 | 3.72E-17 |
| CDKN2A | AL033519.4 | 0.786653927 | 8.35E-33 |
| LIAS | AC022210.1 | 0.603263158 | 3.09E-16 |
| ATP7B | AC068733.1 | 0.64845466 | 2.93E-19 |
| ATP7A | AL353751.1 | 0.607524655 | 1.68E-16 |
| CDKN2A | MIR4713HG | 0.763100119 | 7.48E-30 |
| GLS | AC005034.5 | 0.631676299 | 4.44E-18 |
| SLC31A1 | LINC01270 | 0.686709192 | 3.00E-22 |
| ATP7B | AC021713.1 | 0.717854264 | 4.80E-25 |
| SLC31A1 | FLJ40194 | 0.656684567 | 7.24E-20 |
| GLS | LINC01376 | 0.620955049 | 2.32E-17 |
| DBT | AC022893.1 | 0.620159298 | 2.61E-17 |
| CDKN2A | AC021785.1 | 0.790945786 | 2.21E-33 |
| LIAS | AC007881.4 | 0.610566601 | 1.08E-16 |
| SLC31A1 | LINC00324 | 0.634759953 | 2.73E-18 |
| MTF1 | LINC00324 | 0.627129191 | 9.01E-18 |
| CDKN2A | AL590550.1 | 0.70242686 | 1.29E-23 |
| DBT | AC084824.4 | 0.614983289 | 5.66E-17 |
| ATP7B | AL137789.2 | 0.76055126 | 1.49E-29 |
| ATP7B | AL031599.1 | 0.619141753 | 3.04E-17 |
| DBT | AC019080.1 | 0.63829856 | 1.55E-18 |
| SLC31A1 | LINC02356 | 0.637835935 | 1.67E-18 |
| ATP7B | LINC01217 | 0.797730883 | 2.52E-34 |
| ATP7B | AP000864.1 | 0.600000439 | 4.90E-16 |
| ATP7B | AC010636.2 | 0.620005735 | 2.67E-17 |
| ATP7B | COL18A1-AS1 | 0.813780838 | 1.05E-36 |
| GLS | ANKRD10-IT1 | 0.606505121 | 1.95E-16 |
| ATP7B | AC104561.3 | 0.629290322 | 6.45E-18 |
| CDKN2A | AP001531.1 | 0.791546994 | 1.83E-33 |
| CDKN2A | AP002813.1 | 0.821689758 | 5.78E-38 |
| GLS | AC104596.1 | 0.649700979 | 2.38E-19 |
| CDKN2A | NFE4 | 0.684801088 | 4.34E-22 |
| MTF1 | AC003070.1 | 0.608513785 | 1.46E-16 |
| ATP7B | LINC01218 | 0.816521534 | 3.91E-37 |
| CDKN2A | AL137786.1 | 0.624798056 | 1.29E-17 |
| ATP7B | AC105415.1 | 0.6700091 | 6.87E-21 |
| ATP7A | AC108159.1 | 0.600016276 | 4.89E-16 |
| CDKN2A | AC007391.2 | 0.734113218 | 1.17E-26 |
| ATP7B | LINC01030 | 0.669129618 | 8.05E-21 |
| GLS | AC005261.1 | 0.681220525 | 8.59E-22 |
| DBT | AC005261.1 | 0.62557508 | 1.15E-17 |
| DBT | AC012467.2 | 0.615091131 | 5.57E-17 |
| SLC31A1 | LINC01146 | 0.607876692 | 1.60E-16 |
| ATP7B | AL139294.1 | 0.782198088 | 3.22E-32 |
| GLS | AC010976.1 | 0.602637342 | 3.38E-16 |
| DBT | AC010976.1 | 0.607702557 | 1.64E-16 |
| CDKN2A | AC016629.2 | 0.729737282 | 3.26E-26 |
| ATP7B | AL445309.1 | 0.675107233 | 2.70E-21 |
| GLS | AC107068.1 | 0.622741237 | 1.77E-17 |
| LIAS | ZNF337-AS1 | 0.604557698 | 2.57E-16 |
| GLS | ZNF337-AS1 | 0.659729861 | 4.27E-20 |
| DBT | ZNF337-AS1 | 0.702281828 | 1.33E-23 |
| ATP7B | NLGN1-AS1 | 0.814522707 | 8.05E-37 |
| GLS | AC006008.1 | 0.630895142 | 5.02E-18 |
| ATP7B | AC068759.1 | 0.820625717 | 8.61E-38 |
| CDKN2A | AP005899.2 | 0.793988372 | 8.42E-34 |
| MTF1 | ITPRIP-AS1 | 0.600057586 | 4.86E-16 |
| ATP7B | AC073210.3 | 0.692919244 | 8.87E-23 |
| ATP7A | AC009163.7 | 0.609378789 | 1.29E-16 |
| DBT | AC019080.5 | 0.679124923 | 1.28E-21 |
| GLS | AC008280.2 | 0.702369203 | 1.31E-23 |
| DBT | AC008280.2 | 0.65374344 | 1.20E-19 |
| CDKN2A | AC106793.1 | 0.69616659 | 4.63E-23 |
| SLC31A1 | PTPRN2-AS1 | 0.621826289 | 2.03E-17 |
| MTF1 | PTPRN2-AS1 | 0.605522053 | 2.24E-16 |
| GLS | AC084824.3 | 0.602057691 | 3.67E-16 |
| DBT | AC084824.3 | 0.653845536 | 1.18E-19 |
| ATP7B | MGAT3-AS1 | 0.709809427 | 2.75E-24 |
| DBT | AC022137.3 | 0.613584664 | 6.95E-17 |
| CDKN2A | AC245187.2 | 0.633518545 | 3.32E-18 |
| GLS | AC021851.1 | 0.624518828 | 1.35E-17 |
| SLC31A1 | AL512603.2 | 0.690405908 | 1.46E-22 |
| MTF1 | CFLAR-AS1 | 0.614133816 | 6.41E-17 |
| LIAS | TMEM9B-AS1 | 0.645921665 | 4.46E-19 |
| ATP7B | AC096577.1 | 0.786287197 | 9.35E-33 |
| GLS | WARS2-AS1 | 0.663091386 | 2.37E-20 |
| DBT | WARS2-AS1 | 0.647034317 | 3.71E-19 |
| ATP7B | LINC02770 | 0.795067993 | 5.96E-34 |
| GLS | AC105285.1 | 0.688149587 | 2.27E-22 |
| SLC31A1 | MGC16275 | 0.660770985 | 3.56E-20 |
| ATP7B | LINC00534 | 0.785673353 | 1.13E-32 |
| CDKN2A | AC069155.1 | 0.669818045 | 7.11E-21 |
| ATP7B | AC110011.1 | 0.776214467 | 1.88E-31 |
| ATP7B | AC087369.2 | 0.602947626 | 3.23E-16 |
| NLRP3 | AC010327.8 | 0.6271554 | 8.98E-18 |
| SLC31A1 | AC010327.8 | 0.635857917 | 2.29E-18 |
| GLS | AC093157.1 | 0.603301478 | 3.07E-16 |
| GLS | AL080317.2 | 0.66369216 | 2.13E-20 |
| DBT | AL080317.2 | 0.712995918 | 1.39E-24 |
| ATP7B | LINC00989 | 0.737999191 | 4.61E-27 |
| ATP7B | AC020934.2 | 0.686525569 | 3.11E-22 |
| SLC31A1 | AATBC | 0.611584955 | 9.32E-17 |
| ATP7B | LINC01474 | 0.760758078 | 1.41E-29 |
| ATP7B | AC123912.4 | 0.649775836 | 2.35E-19 |
| GLS | AC078883.1 | 0.656520901 | 7.45E-20 |
| GLS | AC113385.1 | 0.604513422 | 2.59E-16 |
| ATP7B | AL627308.3 | 0.809408125 | 4.92E-36 |
| ATP7B | AC127526.1 | 0.830761493 | 1.73E-39 |
| LIAS | SP2-AS1 | 0.608474125 | 1.47E-16 |
| DBT | SP2-AS1 | 0.697855384 | 3.30E-23 |
| LIAS | STX18-AS1 | 0.61950089 | 2.88E-17 |
| GLS | STX18-AS1 | 0.644221448 | 5.91E-19 |
| DBT | STX18-AS1 | 0.645383912 | 4.88E-19 |
| SLC31A1 | AC024909.1 | 0.647544738 | 3.41E-19 |
| LIAS | AC005670.3 | 0.619626114 | 2.83E-17 |
| GLS | AC005670.3 | 0.693030939 | 8.68E-23 |
| DBT | AC005670.3 | 0.720932434 | 2.42E-25 |
| GLS | AL035448.1 | 0.60938517 | 1.28E-16 |
| CDKN2A | AF127936.1 | 0.710251001 | 2.50E-24 |
| CDKN2A | LINC02201 | 0.791609787 | 1.79E-33 |
| GLS | AC092574.1 | 0.605694938 | 2.19E-16 |
| GLS | AC022272.1 | 0.638276149 | 1.55E-18 |
| CDKN2A | AC073964.1 | 0.743653492 | 1.16E-27 |
| MTF1 | LINC02035 | 0.712840923 | 1.43E-24 |
| GLS | AC011477.1 | 0.654443426 | 1.06E-19 |
| DBT | AC011477.1 | 0.618174759 | 3.52E-17 |
| ATP7B | AL136221.1 | 0.799950147 | 1.22E-34 |
| NLRP3 | AC005280.2 | 0.628709408 | 7.06E-18 |
| SLC31A1 | AC005280.2 | 0.704065369 | 9.21E-24 |
| MTF1 | AC005280.2 | 0.652876486 | 1.39E-19 |
| ATP7B | AC123788.1 | 0.683174821 | 5.92E-22 |
| ATP7B | BOLA3-AS1 | 0.748207551 | 3.71E-28 |
| SLC31A1 | AC138207.9 | 0.733647595 | 1.30E-26 |
| GLS | FIRRE | 0.623822412 | 1.50E-17 |
| GCSH | FIRRE | 0.626926961 | 9.30E-18 |
| LIAS | AC010834.3 | 0.610566798 | 1.08E-16 |
| GLS | AC010834.3 | 0.623771984 | 1.51E-17 |
| DBT | AC010834.3 | 0.628909327 | 6.84E-18 |
| DBT | NORAD | 0.679872144 | 1.11E-21 |
| ATP7A | AC007390.1 | 0.612442205 | 8.22E-17 |
| GLS | AC007390.1 | 0.624547074 | 1.34E-17 |
| DBT | AC007390.1 | 0.687021869 | 2.82E-22 |
| ATP7B | LINC00871 | 0.707479351 | 4.50E-24 |
| CDKN2A | AC116049.1 | 0.793591828 | 9.55E-34 |
| GLS | IQCH-AS1 | 0.630022542 | 5.75E-18 |
| LIAS | AL354696.2 | 0.630578934 | 5.27E-18 |
| DBT | AL354696.2 | 0.650681419 | 2.02E-19 |
| CDKN2A | AC127526.5 | 0.692418816 | 9.80E-23 |
| ATP7B | NOL4L-DT | 0.792906416 | 1.19E-33 |
| GLS | KTN1-AS1 | 0.617800522 | 3.72E-17 |
| ATP7B | AC104071.1 | 0.777170106 | 1.42E-31 |
| CDKN2A | AC106900.1 | 0.772253734 | 5.87E-31 |
| SLC31A1 | LINC02528 | 0.602072151 | 3.66E-16 |
| CDKN2A | CDKN2B-AS1 | 0.667815149 | 1.02E-20 |
| ATP7A | AL157786.1 | 0.631014098 | 4.92E-18 |
| DBT | AL157786.1 | 0.64042983 | 1.10E-18 |
| ATP7B | LINC01151 | 0.786975601 | 7.57E-33 |
| LIAS | CHKB-DT | 0.620656506 | 2.42E-17 |
| GLS | AC004076.2 | 0.670676062 | 6.08E-21 |
| DBT | AC004076.2 | 0.625914325 | 1.09E-17 |
| GLS | LINC00624 | 0.702699477 | 1.22E-23 |
| DBT | LINC00624 | 0.656682577 | 7.25E-20 |
| GLS | LINC00662 | 0.609897884 | 1.19E-16 |
| DBT | LINC00662 | 0.604441561 | 2.61E-16 |
| ATP7B | AC092118.1 | 0.661712504 | 3.02E-20 |
| ATP7B | AL353689.3 | 0.748882849 | 3.12E-28 |
| LIAS | AC015849.3 | 0.733805923 | 1.26E-26 |
| ATP7B | BX322562.1 | 0.743391226 | 1.24E-27 |
| GCSH | AC024896.1 | 0.627159557 | 8.97E-18 |
| GLS | AL080317.1 | 0.678447726 | 1.45E-21 |
| DBT | AL080317.1 | 0.716617832 | 6.30E-25 |
| ATP7B | AC127526.2 | 0.637848942 | 1.66E-18 |
| CDKN2A | AC127526.2 | 0.676114749 | 2.24E-21 |
| GLS | FBXO30-DT | 0.636386465 | 2.10E-18 |
| DBT | FBXO30-DT | 0.643697087 | 6.44E-19 |
| SLC31A1 | AC105265.2 | 0.728546704 | 4.29E-26 |
| ATP7B | AC015802.7 | 0.778035268 | 1.11E-31 |
| ATP7B | AL031848.1 | 0.609477731 | 1.27E-16 |
| CDKN2A | LINC01340 | 0.777770576 | 1.20E-31 |
| CDKN2A | AC018742.1 | 0.701578574 | 1.54E-23 |
| ATP7B | PLAC4 | 0.738846808 | 3.76E-27 |
| ATP7B | AL118508.1 | 0.669799013 | 7.13E-21 |
| GLS | AP001893.1 | 0.641212743 | 9.66E-19 |
| DBT | AP001893.1 | 0.613335114 | 7.21E-17 |
| GLS | LINC02062 | 0.601464455 | 3.99E-16 |
| GLS | EBLN3P | 0.786040037 | 1.01E-32 |
| DBT | EBLN3P | 0.657219129 | 6.61E-20 |
| GLS | AC092645.2 | 0.608049273 | 1.56E-16 |
| ATP7B | MIR3681HG | 0.7338774 | 1.23E-26 |
| ATP7B | AC004053.1 | 0.647461442 | 3.46E-19 |
| LIAS | ZNF32-AS1 | 0.609665968 | 1.23E-16 |
| ATP7B | AC010378.1 | 0.702567755 | 1.26E-23 |
| GLS | ZBED3-AS1 | 0.672382774 | 4.46E-21 |
| DBT | ZBED3-AS1 | 0.635969262 | 2.25E-18 |
| SLC31A1 | AC242988.2 | 0.628366185 | 7.44E-18 |
| ATP7B | AL355432.1 | 0.739610354 | 3.12E-27 |
| GLS | AC011477.2 | 0.679346565 | 1.22E-21 |
| DBT | AC011477.2 | 0.689773102 | 1.65E-22 |
| LIAS | AC068631.1 | 0.664436464 | 1.87E-20 |
| DBT | AL354696.1 | 0.611823949 | 9.00E-17 |
| ATP7B | LINC01014 | 0.764482742 | 5.13E-30 |
| ATP7B | GATA2-AS1 | 0.622660166 | 1.79E-17 |
| GLS | LRRC8C-DT | 0.624811918 | 1.29E-17 |
| LIAS | ELOA-AS1 | 0.64444878 | 5.69E-19 |
| DBT | ELOA-AS1 | 0.623032595 | 1.69E-17 |
| SLC31A1 | AC010247.1 | 0.64165226 | 9.00E-19 |
| ATP7B | SMILR | 0.760380669 | 1.56E-29 |
| MTF1 | AC025280.3 | 0.602650851 | 3.37E-16 |
| DBT | RTCA-AS1 | 0.690247108 | 1.50E-22 |
| DBT | MCPH1-AS1 | 0.600841027 | 4.35E-16 |
| ATP7B | AC079776.5 | 0.741667418 | 1.89E-27 |
| MTF1 | AC006238.2 | 0.608665812 | 1.43E-16 |
| ATP7B | AC103876.1 | 0.60716172 | 1.77E-16 |
| CDKN2A | LINC02234 | 0.792664352 | 1.28E-33 |
| CDKN2A | AP003498.2 | 0.770678379 | 9.17E-31 |
| ATP7B | AC007405.2 | 0.844465182 | 5.73E-42 |
| SLC31A1 | LRP1-AS | 0.628188874 | 7.65E-18 |
| LIAS | AC020978.1 | 0.637762775 | 1.69E-18 |
| ATP7A | AC099811.4 | 0.607887071 | 1.60E-16 |
| GLS | AC099811.4 | 0.629464259 | 6.27E-18 |
| GCSH | AC092718.1 | 0.652928102 | 1.38E-19 |
| SLC31A1 | C2-AS1 | 0.614332237 | 6.23E-17 |
| GLS | AC026356.1 | 0.699626465 | 2.30E-23 |
| DBT | AC026356.1 | 0.657315225 | 6.50E-20 |
| ATP7B | AC008736.1 | 0.651110198 | 1.87E-19 |
| DBT | AC007406.4 | 0.649939072 | 2.28E-19 |
| ATP7B | AL133499.1 | 0.601954822 | 3.72E-16 |
| ATP7B | AC002069.2 | 0.743771807 | 1.12E-27 |
| ATP7B | LINC01366 | 0.758383989 | 2.65E-29 |
| GLS | TTC28-AS1 | 0.620260131 | 2.57E-17 |
| CDKN2A | AC003093.1 | 0.783114345 | 2.45E-32 |
| DBT | AC006213.4 | 0.633286648 | 3.44E-18 |
| ATP7A | SDCBP2-AS1 | 0.600224964 | 4.75E-16 |
| GLS | SDCBP2-AS1 | 0.600837769 | 4.36E-16 |
| DBT | SDCBP2-AS1 | 0.606752481 | 1.88E-16 |
| SLC31A1 | PELATON | 0.717775773 | 4.89E-25 |
| ATP7B | AC113133.1 | 0.634249979 | 2.96E-18 |
| CDKN2A | RIPOR3-AS1 | 0.640435671 | 1.10E-18 |
| GLS | AC090241.2 | 0.629315747 | 6.42E-18 |
| ATP7B | C15orf54 | 0.710291513 | 2.48E-24 |
| LIAS | AC068025.1 | 0.648528316 | 2.89E-19 |
| GLS | AC068025.1 | 0.627706352 | 8.24E-18 |
| DBT | AC068025.1 | 0.623179647 | 1.65E-17 |
| GLS | C21orf62-AS1 | 0.602466481 | 3.46E-16 |
| DBT | C21orf62-AS1 | 0.628190857 | 7.65E-18 |
| DBT | NUTM2A-AS1 | 0.60406122 | 2.76E-16 |
| ATP7B | AC013474.2 | 0.739130508 | 3.51E-27 |
| GLS | AC040934.1 | 0.649347504 | 2.52E-19 |
| ATP7B | AL499627.2 | 0.744716934 | 8.89E-28 |
| LIPT1 | DNAJC3-DT | 0.626040364 | 1.07E-17 |
| GLS | FTX | 0.76999161 | 1.11E-30 |
| DBT | FTX | 0.710949827 | 2.15E-24 |
| ATP7B | AC105219.1 | 0.628659216 | 7.11E-18 |
| ATP7B | AC084864.2 | 0.649102973 | 2.63E-19 |
| GLS | AC098484.4 | 0.609468408 | 1.27E-16 |
| DBT | AC093227.3 | 0.640156665 | 1.15E-18 |
| ATP7B | AP001189.3 | 0.646656788 | 3.95E-19 |
| SLC31A1 | AC138207.4 | 0.658634375 | 5.17E-20 |
| MTF1 | AC138207.4 | 0.610576745 | 1.08E-16 |
| LIAS | AC025682.1 | 0.612935399 | 7.65E-17 |
| SLC31A1 | AC007728.3 | 0.665320406 | 1.60E-20 |
| MTF1 | AC007728.3 | 0.610684894 | 1.06E-16 |
| GLS | PRKCQ-AS1 | 0.622563505 | 1.81E-17 |
| SLC31A1 | AP005059.2 | 0.600139036 | 4.81E-16 |
| ATP7B | AC005009.1 | 0.651466587 | 1.77E-19 |
| SLC31A1 | AC008055.2 | 0.665813666 | 1.46E-20 |
| ATP7A | Z83843.1 | 0.603439915 | 3.02E-16 |
| GLS | Z83843.1 | 0.756773078 | 4.06E-29 |
| DBT | Z83843.1 | 0.728365087 | 4.48E-26 |
| CDKN2A | AF121898.1 | 0.665370836 | 1.58E-20 |
| MTF1 | LUCAT1 | 0.606402823 | 1.97E-16 |
| GLS | AC078785.1 | 0.623721598 | 1.52E-17 |
| ATP7B | AP001528.1 | 0.620814746 | 2.37E-17 |
| DBT | NNT-AS1 | 0.608578239 | 1.44E-16 |
| CDKN2A | AL133338.1 | 0.627443229 | 8.59E-18 |
| DBT | AL731563.3 | 0.673513422 | 3.62E-21 |
| SLC31A1 | B4GALT1-AS1 | 0.656172762 | 7.91E-20 |
| NLRP3 | AL929091.1 | 0.633811123 | 3.17E-18 |
| SLC31A1 | KLHDC7B-DT | 0.612277942 | 8.42E-17 |
| ATP7B | AC024361.1 | 0.69400469 | 7.15E-23 |
| CDKN2A | AL161663.2 | 0.633309238 | 3.43E-18 |
| GLS | AL021368.2 | 0.686787082 | 2.96E-22 |
| DBT | AL021368.2 | 0.648577344 | 2.87E-19 |
| NLRP3 | LINC02705 | 0.685919936 | 3.50E-22 |
| SLC31A1 | LINC02705 | 0.631954289 | 4.25E-18 |
| ATP7A | AL022322.2 | 0.61661235 | 4.44E-17 |
| SLC31A1 | AC087741.1 | 0.603392269 | 3.04E-16 |
| CDKN2A | AP003498.1 | 0.776605323 | 1.68E-31 |
| GLS | AC026356.2 | 0.621616278 | 2.10E-17 |
| ATP7B | MAGI1-IT1 | 0.625419763 | 1.17E-17 |
| CDKN2A | MAGI1-IT1 | 0.677968702 | 1.58E-21 |
| ATP7B | TSBP1-AS1 | 0.750757215 | 1.94E-28 |
| DLST | MRPL20-AS1 | 0.657054619 | 6.80E-20 |
| GLS | AC060780.1 | 0.611792976 | 9.04E-17 |
| ATP7B | AC064869.1 | 0.71647785 | 6.50E-25 |
| DBT | AL008729.1 | 0.625559023 | 1.15E-17 |
| ATP7B | AC026782.2 | 0.618262359 | 3.47E-17 |
| LIAS | LACTB2-AS1 | 0.62065439 | 2.42E-17 |
| DBT | AF287957.1 | 0.614850199 | 5.77E-17 |
| MTF1 | AC000403.1 | 0.613577594 | 6.96E-17 |
| ATP7B | AL356747.1 | 0.764456643 | 5.17E-30 |
| ATP7B | AL450344.2 | 0.682761399 | 6.41E-22 |
| SLC31A1 | LINC00877 | 0.712515639 | 1.54E-24 |
| SLC31A1 | AC245884.11 | 0.67396669 | 3.33E-21 |
| ATP7B | AP002761.2 | 0.752555811 | 1.22E-28 |
| DBT | AC107027.3 | 0.623398114 | 1.60E-17 |
| LIAS | UBE2D3-AS1 | 0.694838726 | 6.05E-23 |
| ATP7B | AC010615.2 | 0.648548301 | 2.88E-19 |
| MTF1 | ARAP1-AS2 | 0.646692614 | 3.93E-19 |
| CDKN2A | AC106799.3 | 0.790947326 | 2.20E-33 |
| MTF1 | AC008753.2 | 0.637019495 | 1.90E-18 |
| ATP7B | AC092542.1 | 0.612111054 | 8.63E-17 |
| ATP7B | AC018685.3 | 0.617344522 | 3.98E-17 |
| ATP7B | AC129492.1 | 0.65515382 | 9.43E-20 |
| ATP7B | LINC01216 | 0.737527786 | 5.16E-27 |
| ATP7B | AC093766.1 | 0.826196943 | 1.04E-38 |
| SLC31A1 | AL627309.5 | 0.630820303 | 5.08E-18 |
| DBT | INTS6-AS1 | 0.607441712 | 1.70E-16 |
| LIAS | HCG25 | 0.676241905 | 2.19E-21 |
| GLS | AL357552.2 | 0.602818309 | 3.29E-16 |
| GLS | AC006460.2 | 0.623330386 | 1.61E-17 |
| ATP7B | LINC02151 | 0.713012124 | 1.38E-24 |
| DBT | LIMS1-AS1 | 0.616402529 | 4.58E-17 |
| CDKN2A | AC079148.3 | 0.726624966 | 6.68E-26 |
| GLS | BACE1-AS | 0.628582794 | 7.20E-18 |
| DBT | BACE1-AS | 0.69722789 | 3.74E-23 |
| GLS | Z68871.1 | 0.638183251 | 1.58E-18 |
| DBT | Z68871.1 | 0.650985516 | 1.91E-19 |
| DBT | AL356019.2 | 0.622335644 | 1.88E-17 |
| SLC31A1 | AC133919.1 | 0.613876845 | 6.66E-17 |
| ATP7B | AC007786.2 | 0.605788512 | 2.16E-16 |
| DLST | TONSL-AS1 | 0.628876728 | 6.87E-18 |
| ATP7A | MCM3AP-AS1 | 0.611863037 | 8.95E-17 |
| LIAS | MCM3AP-AS1 | 0.603116147 | 3.16E-16 |
| GLS | MCM3AP-AS1 | 0.680410486 | 1.00E-21 |
| DBT | MCM3AP-AS1 | 0.715596014 | 7.88E-25 |
| LIAS | AC022400.4 | 0.60066242 | 4.46E-16 |
| DBT | AC022400.4 | 0.604245111 | 2.69E-16 |
| DBT | AL132989.1 | 0.648253293 | 3.03E-19 |
| MTF1 | LINC01547 | 0.627417355 | 8.62E-18 |
| ATP7B | AC099329.1 | 0.804557566 | 2.60E-35 |
| DBT | USP46-DT | 0.602917224 | 3.25E-16 |
| GLS | AF117829.1 | 0.65621611 | 7.85E-20 |
| GLS | LINC02100 | 0.602621726 | 3.39E-16 |
| CDKN2A | FGF12-AS3 | 0.662702124 | 2.54E-20 |
| ATP7B | AC100801.1 | 0.660494066 | 3.74E-20 |
| ATP7A | SOS1-IT1 | 0.699076804 | 2.57E-23 |
| GLS | SOS1-IT1 | 0.626835194 | 9.43E-18 |
| DBT | SOS1-IT1 | 0.642419151 | 7.94E-19 |
| GLS | NIPBL-DT | 0.685327643 | 3.92E-22 |
| DBT | NIPBL-DT | 0.630861604 | 5.04E-18 |
| GLS | AC096586.2 | 0.694599155 | 6.35E-23 |
| SLC31A1 | AC138207.1 | 0.711226583 | 2.03E-24 |
| MTF1 | AC138207.1 | 0.619337772 | 2.95E-17 |
| GLS | N4BP2L2-IT2 | 0.605564707 | 2.23E-16 |
| DBT | N4BP2L2-IT2 | 0.633846451 | 3.15E-18 |
| ATP7B | MAGI1-AS1 | 0.689747317 | 1.66E-22 |
| DBT | AC006213.5 | 0.637321602 | 1.81E-18 |
| ATP7B | AL132982.1 | 0.724725304 | 1.03E-25 |
| GLS | PSMA3-AS1 | 0.640802238 | 1.03E-18 |
| DBT | PSMA3-AS1 | 0.61124106 | 9.80E-17 |
| ATP7B | AL138716.1 | 0.774901956 | 2.75E-31 |
| GLS | AL096701.3 | 0.604639269 | 2.54E-16 |
| DBT | AL096701.3 | 0.618201556 | 3.50E-17 |
| CDKN2A | AC010809.3 | 0.690344315 | 1.48E-22 |
| CDKN2A | AC017006.1 | 0.690777749 | 1.36E-22 |
| CDKN2A | LINC00520 | 0.715075076 | 8.83E-25 |
| GLS | MANEA-DT | 0.626277627 | 1.03E-17 |
| DBT | MANEA-DT | 0.621228494 | 2.22E-17 |
| CDKN2A | AL592156.1 | 0.609889519 | 1.19E-16 |
| ATP7A | AL512353.1 | 0.60115056 | 4.17E-16 |
| GLS | AP001469.2 | 0.637914658 | 1.65E-18 |
| DBT | AP001469.2 | 0.631743247 | 4.39E-18 |
| ATP7A | AC099811.5 | 0.680453171 | 9.93E-22 |
| GLS | AC099811.5 | 0.666278244 | 1.34E-20 |
| DBT | AC099811.5 | 0.636536299 | 2.05E-18 |
| ATP7B | AC009486.2 | 0.614474556 | 6.10E-17 |
| CDKN2A | LINC02315 | 0.703330951 | 1.07E-23 |
| GLS | SMYD3-IT1 | 0.610987601 | 1.02E-16 |
| SLC31A1 | AP002954.1 | 0.64122188 | 9.65E-19 |
| GLS | AL160400.1 | 0.665497439 | 1.55E-20 |
| ATP7B | AC078850.2 | 0.733482649 | 1.36E-26 |
| GLS | ZNF433-AS1 | 0.628410223 | 7.39E-18 |
| CDKN2A | CHRM3-AS2 | 0.78774606 | 5.97E-33 |
| GLS | COX10-AS1 | 0.612793481 | 7.81E-17 |
| DBT | COX10-AS1 | 0.670631178 | 6.13E-21 |
| MTF1 | AC104118.1 | 0.633673769 | 3.24E-18 |
| ATP7B | AC090409.1 | 0.709100818 | 3.19E-24 |
| SLC31A1 | AL157871.2 | 0.677178543 | 1.84E-21 |
| ATP7B | AL138916.1 | 0.73965033 | 3.09E-27 |
| ATP7A | AC025917.1 | 0.659856916 | 4.18E-20 |
| GLS | AC025917.1 | 0.708893681 | 3.34E-24 |
| DBT | AC025917.1 | 0.645328415 | 4.92E-19 |
| CDKN2A | AC079148.2 | 0.749348778 | 2.77E-28 |
| ATP7B | AC100834.2 | 0.79831496 | 2.08E-34 |
| ATP7B | PAPPA-AS2 | 0.72051485 | 2.66E-25 |
| ATP7B | AL162413.1 | 0.835243758 | 2.84E-40 |
| ATP7B | PBX1-AS1 | 0.67382543 | 3.42E-21 |
| SLC31A1 | AC121338.1 | 0.665482852 | 1.55E-20 |
| GLS | GMDS-DT | 0.625086965 | 1.23E-17 |
| DBT | GMDS-DT | 0.670823777 | 5.92E-21 |
| CDKN2A | AC108058.1 | 0.694710711 | 6.21E-23 |
| ATP7B | LINC02412 | 0.707692542 | 4.30E-24 |
| GLS | AC020612.3 | 0.653369106 | 1.28E-19 |
| DBT | AC020612.3 | 0.663733713 | 2.11E-20 |
| LIAS | ZNF32-AS2 | 0.616159982 | 4.75E-17 |
| ATP7B | AC093297.1 | 0.608569176 | 1.45E-16 |
| CDKN2A | AL162464.2 | 0.730526934 | 2.71E-26 |
| CDKN2A | LINC02156 | 0.748569972 | 3.38E-28 |
| SLC31A1 | AC138207.5 | 0.708261328 | 3.82E-24 |
| GLS | AL355488.1 | 0.605011185 | 2.41E-16 |
| CDKN2A | AL121584.1 | 0.632982997 | 3.61E-18 |
| ATP7B | AL358232.2 | 0.729527405 | 3.42E-26 |
| NLRP3 | LINC01503 | 0.610512864 | 1.09E-16 |
| SLC31A1 | LINC01503 | 0.788985163 | 4.07E-33 |
| GLS | AL353796.1 | 0.60828809 | 1.51E-16 |
| DBT | AL353796.1 | 0.610628194 | 1.07E-16 |
| CDKN2A | AC236668.1 | 0.640147384 | 1.15E-18 |
| ATP7B | LINC02232 | 0.725840954 | 8.00E-26 |
| CDKN2A | AL035409.1 | 0.628779447 | 6.98E-18 |
| DBT | LYRM4-AS1 | 0.600933098 | 4.30E-16 |
| ATP7A | AC099811.1 | 0.644737514 | 5.43E-19 |
| GLS | AC099811.1 | 0.661446113 | 3.17E-20 |
| GLS | AL512343.2 | 0.613932606 | 6.61E-17 |
| DBT | DLEU2L | 0.606190183 | 2.04E-16 |
| GLS | AC011815.2 | 0.6219886 | 1.98E-17 |
| DBT | AC011815.2 | 0.628329884 | 7.48E-18 |
| SLC31A1 | AC007406.1 | 0.635672605 | 2.36E-18 |
| ATP7B | AC069410.1 | 0.804252413 | 2.89E-35 |
| GLS | AL359715.1 | 0.687837856 | 2.41E-22 |
| NLRP3 | MIR22HG | 0.601889385 | 3.76E-16 |
| GLS | AL021918.5 | 0.647252988 | 3.58E-19 |
| DBT | AL021918.5 | 0.610626038 | 1.07E-16 |
| ATP7B | AL022396.1 | 0.755091169 | 6.32E-29 |
| SLC31A1 | AC215522.2 | 0.664874265 | 1.73E-20 |
| ATP7B | COL18A1-AS2 | 0.759285007 | 2.09E-29 |
| CDKN2A | AC004691.1 | 0.644221906 | 5.91E-19 |
| SLC31A1 | AC002451.1 | 0.628812166 | 6.94E-18 |
| CDKN2A | AC100801.2 | 0.788028955 | 5.47E-33 |
| CDKN2A | AL592424.1 | 0.742670467 | 1.48E-27 |
| NLRP3 | AC005632.3 | 0.61509352 | 5.56E-17 |
| SLC31A1 | PSMB8-AS1 | 0.616188157 | 4.73E-17 |
| CDKN2A | SLC25A21-AS1 | 0.701576083 | 1.54E-23 |
| GLS | CSTF3-DT | 0.652191677 | 1.56E-19 |
| GLS | LINC00216 | 0.60682774 | 1.86E-16 |
| ATP7B | AC009971.1 | 0.715949854 | 7.30E-25 |
| ATP7B | LINC02046 | 0.796603396 | 3.63E-34 |
| DBT | PAXBP1-AS1 | 0.601813811 | 3.80E-16 |
| GLS | NIFK-AS1 | 0.646942387 | 3.77E-19 |
| DBT | NIFK-AS1 | 0.674252411 | 3.16E-21 |
| GLS | CKMT2-AS1 | 0.646229469 | 4.24E-19 |
| DBT | CKMT2-AS1 | 0.683976976 | 5.08E-22 |
| ATP7B | MYO16-AS2 | 0.723819269 | 1.27E-25 |
| GLS | ERICH6-AS1 | 0.606932451 | 1.83E-16 |
| SLC31A1 | AC005071.1 | 0.660856092 | 3.51E-20 |
| MTF1 | AC005071.1 | 0.639049768 | 1.37E-18 |
| DBT | AC000120.1 | 0.602438837 | 3.47E-16 |
| ATP7B | AL109837.3 | 0.733339036 | 1.40E-26 |
| GLS | AC090241.3 | 0.618324059 | 3.44E-17 |
| LIAS | AC009120.2 | 0.646957704 | 3.76E-19 |
| GLS | AC009120.2 | 0.606463947 | 1.96E-16 |
| DBT | AC009120.2 | 0.657305705 | 6.51E-20 |
| NLRP3 | SRGAP2-AS1 | 0.604731 | 2.51E-16 |
| GLS | AL161756.1 | 0.600427167 | 4.62E-16 |
| ATP7B | AC008554.1 | 0.676210147 | 2.20E-21 |
| ATP7B | LINC01033 | 0.748108437 | 3.80E-28 |
| GLS | LINC01355 | 0.631920943 | 4.27E-18 |
| DBT | AP001432.1 | 0.65529618 | 9.20E-20 |
| GLS | AC026741.1 | 0.615791226 | 5.02E-17 |
| DLST | ANAPC1P2 | 0.605185253 | 2.35E-16 |
| ATP7B | AC025741.1 | 0.696854928 | 4.03E-23 |
| LIAS | AC107214.1 | 0.624340147 | 1.38E-17 |
| ATP7A | AC110611.1 | 0.602139307 | 3.63E-16 |
| DBT | AC110611.1 | 0.64135622 | 9.44E-19 |
| GLS | HCG18 | 0.659705069 | 4.29E-20 |
| DBT | HCG18 | 0.687142767 | 2.76E-22 |
| CDKN2A | AC079466.1 | 0.809273216 | 5.16E-36 |
| ATP7B | THORLNC | 0.621897491 | 2.01E-17 |
| DLST | AL360181.2 | 0.611698883 | 9.17E-17 |
| GLS | AC022364.1 | 0.613344985 | 7.20E-17 |
| ATP7B | LINC01133 | 0.614740592 | 5.86E-17 |
| CDKN2A | LINC01133 | 0.702764251 | 1.21E-23 |
| CDKN2A | GPR1-AS | 0.777078816 | 1.46E-31 |
| MTF1 | AL035588.1 | 0.628019181 | 7.85E-18 |
| ATP7B | AC093159.1 | 0.759072175 | 2.21E-29 |
| ATP7B | AL360015.1 | 0.67552115 | 2.50E-21 |
| GLS | AC104335.1 | 0.661971611 | 2.89E-20 |
| ATP7B | AC004083.1 | 0.800618599 | 9.75E-35 |
| ATP7B | AC108136.1 | 0.674174066 | 3.21E-21 |
| CDKN2A | AL035409.2 | 0.739846223 | 2.95E-27 |
| MTF1 | AL121933.2 | 0.66519628 | 1.63E-20 |
| ATP7A | HHLA3-AS1 | 0.602643049 | 3.38E-16 |
| DBT | HHLA3-AS1 | 0.634489495 | 2.85E-18 |
| MTF1 | AP003065.1 | 0.667677536 | 1.05E-20 |
| LIAS | AC093297.2 | 0.630163871 | 5.63E-18 |
| GLS | AC093297.2 | 0.671651419 | 5.09E-21 |
| DBT | AC093297.2 | 0.669937325 | 6.96E-21 |
| GLS | TSC22D1-AS1 | 0.600674213 | 4.46E-16 |
| CDKN2A | AC104407.1 | 0.791517563 | 1.84E-33 |
| ATP7B | LINC02606 | 0.604521359 | 2.58E-16 |
| GLS | AC010320.4 | 0.605202082 | 2.35E-16 |
| SLC31A1 | C17orf77 | 0.671513205 | 5.22E-21 |
| GLS | AC009107.2 | 0.62147833 | 2.14E-17 |
| DBT | AC009107.2 | 0.620343763 | 2.54E-17 |
| GLS | AC092747.4 | 0.616851608 | 4.29E-17 |
| DBT | AC026979.4 | 0.638523692 | 1.49E-18 |
| LIPT2 | AP001372.2 | 0.60414769 | 2.73E-16 |
| CDKN2A | AL139002.1 | 0.791855708 | 1.66E-33 |
| ATP7B | AL391001.1 | 0.741334959 | 2.05E-27 |
| MTF1 | AC079174.2 | 0.633928769 | 3.11E-18 |
| LIAS | OIP5-AS1 | 0.625652632 | 1.13E-17 |
| GLS | OIP5-AS1 | 0.645770139 | 4.58E-19 |
| DBT | OIP5-AS1 | 0.746603665 | 5.55E-28 |
| GLS | AL596202.1 | 0.666012658 | 1.41E-20 |
| DBT | AL596202.1 | 0.660923061 | 3.47E-20 |
| CDKN2A | LINC00839 | 0.758544592 | 2.54E-29 |
| SLC31A1 | AC069209.2 | 0.688628184 | 2.07E-22 |
| SLC31A1 | AC007728.2 | 0.668046648 | 9.79E-21 |
| MTF1 | AC007728.2 | 0.655441331 | 8.97E-20 |
| ATP7B | AC079804.3 | 0.679743451 | 1.14E-21 |
| GLS | AC005519.1 | 0.628925099 | 6.82E-18 |
| DBT | AC005519.1 | 0.628322487 | 7.49E-18 |
| GLS | AC242426.2 | 0.652265233 | 1.54E-19 |
| NLRP3 | LINC00968 | 0.64071213 | 1.05E-18 |
| SLC31A1 | LINC00968 | 0.662604146 | 2.58E-20 |
| MTF1 | LINC00968 | 0.642151099 | 8.29E-19 |
| GLS | AL359715.2 | 0.680064291 | 1.07E-21 |
| CDKN2A | AC010261.2 | 0.677421717 | 1.76E-21 |
