# Supplementary material for: Cuproptosis-related lncRNA signature for prognostic prediction in patients with acute myeloid leukemia
Source: BMC Bioinformatics. 2023 Feb 3;24:37. doi: 10.1186/s12859-023-05148-9 (PMC9896718; doi:10.1186/s12859-023-05148-9)
Supplement: Supplementary file 3 — Additional file 3. Table S3 Regression coefficients of cuproptosis-related lncRNAs determined via multi-Cox analysis. [file 12859_2023_5148_MOESM3_ESM.docx]

**Supplementary Table S3. Regression coefficients of cuproptosis-related lncRNAs determined via multi-Cox analysis**

| **Gene** | **coef** |
| --- | --- |
| NFE4 | 0.65782196 |
| LINC00989 | 0.46133181 |
| LINC02062 | -1.257216227 |
| AC006460.2 | 2.002381522 |
| AL353796.1 | -1.264004754 |
| PSMB8-AS1` | 0.774944396 |
| AC000120.1 | -0.959559384 |
