# Supplementary material for: Cuproptosis-related lncRNA signature for prognostic prediction in patients with acute myeloid leukemia
Source: BMC Bioinformatics. 2023 Feb 3;24:37. doi: 10.1186/s12859-023-05148-9 (PMC9896718; doi:10.1186/s12859-023-05148-9)
Supplement: Supplementary file 4 — Additional file 4. Table S4 Differential genes between the high- and low-risk groups. [file 12859_2023_5148_MOESM4_ESM.docx]

**Supplementary Table S4. Differential genes between the high- and low-risk groups**

| **Gene** | **lowMean** | **highMean** | **logFC** | **p** | **fdr** |
| --- | --- | --- | --- | --- | --- |
| AC005153.1 | 2.79E+00 | 1.257830769 | -1.151740244 | 4.42E-05 | 0.0008804 |
| MAL | 4.20E+00 | 12.59084769 | 1.582709656 | 0.0006628 | 0.0048778 |
| IRX5 | 6.25E+00 | 1.241512308 | -2.332731156 | 0.0023444 | 0.0116195 |
| TGM5 | 4.62E+00 | 2.270893846 | -1.025354847 | 0.0011781 | 0.0071674 |
| MTND3P12 | 1.54E+00 | 4.140446154 | 1.427841141 | 0.0120135 | 0.0364405 |
| RN7SKP112 | 1.64E+00 | 0.576949231 | -1.503938871 | 2.46E-05 | 0.0006408 |
| GNG11 | 3.18E+00 | 7.742764615 | 1.285052377 | 0.0010302 | 0.0065112 |
| AC100854.1 | 0.885624324 | 2.01586 | 1.19E+00 | 0.0010005 | 0.0063896 |
| AC006511.4 | 2.068408108 | 1.001958462 | -1.05E+00 | 0.0002505 | 0.0025782 |
| CYTL1 | 293.3659622 | 88.62243538 | -1.73E+00 | 0.0006835 | 0.0049803 |
| SYTL4 | 2.126187838 | 4.816856923 | 1.18E+00 | 9.68E-05 | 0.0014288 |
| SCML2P2 | 26.97308649 | 12.51056308 | -1.11E+00 | 2.25E-07 | 6.16E-05 |
| VNN2 | 15.03033378 | 56.63313385 | 1.91E+00 | 1.03E-06 | 0.0001179 |
| AJ239328.1 | 2.912772973 | 0.831872308 | -1.81E+00 | 0.0022526 | 0.011303 |
| PDE2A-AS2 | 0.963044595 | 2.342470769 | 1.28E+00 | 5.05E-07 | 8.22E-05 |
| C11orf97 | 2.057372973 | 1.002230769 | -1.04E+00 | 0.0003853 | 0.0034334 |
| AC124798.2 | 5.300825676 | 2.138110769 | -1.31E+00 | 1.67E-10 | 3.75E-06 |
| AC244502.3 | 31.91948243 | 14.17916308 | -1.17E+00 | 1.59E-05 | 0.0004976 |
| C1QB | 14.91229865 | 82.80504769 | 2.47E+00 | 0.014099 | 0.040971 |
| RN7SL141P | 23.3362 | 11.51269077 | -1.02E+00 | 4.16E-06 | 0.0002454 |
| GADD45G | 0.968610811 | 2.344575385 | 1.28E+00 | 3.30E-05 | 0.0007543 |
| AL032821.1 | 1.54187027 | 4.484473846 | 1.54E+00 | 0.0001256 | 0.0016854 |
| CD40 | 4.737617568 | 9.922676923 | 1.07E+00 | 3.36E-05 | 0.000759 |
| HTR7 | 2.257395946 | 5.149715385 | 1.19E+00 | 6.24E-05 | 0.0010997 |
| AC090206.1 | 3.175948649 | 1.329572308 | -1.26E+00 | 7.96E-05 | 0.0012664 |
| PLAAT5 | 2.786717568 | 0.624767692 | -2.16E+00 | 0.0123731 | 0.0371472 |
| F12 | 1.723341892 | 3.655669231 | 1.08E+00 | 0.000361 | 0.003294 |
| LINC01871 | 4.187917568 | 8.456092308 | 1.01E+00 | 0.0003223 | 0.0030373 |
| UGT3A2 | 26.41050811 | 4.158709231 | -2.67E+00 | 0.0010514 | 0.0066168 |
| LPO | 48.74195811 | 15.43930615 | -1.66E+00 | 5.30E-05 | 0.0009902 |
| RN7SKP40 | 2.170036486 | 0.955730769 | -1.18E+00 | 0.0002453 | 0.0025475 |
| FTH1P8 | 5.558290541 | 11.76171385 | 1.08E+00 | 1.21E-05 | 0.0004226 |
| CDC42EP1 | 3.450866216 | 9.443396923 | 1.45E+00 | 6.45E-05 | 0.0011194 |
| CD300E | 30.02241486 | 91.45660154 | 1.61E+00 | 5.80E-05 | 0.0010543 |
| AC097504.2 | 0.935318919 | 3.86926 | 2.05E+00 | 0.0021516 | 0.0109364 |
| RPS27AP8 | 20.37745946 | 9.594347692 | -1.09E+00 | 1.36E-06 | 0.0001405 |
| AGAP9 | 41.15027027 | 17.97857692 | -1.19E+00 | 0.0061896 | 0.0227833 |
| HLA-DQB2 | 6.130925676 | 13.17564 | 1.10E+00 | 0.0002016 | 0.0022713 |
| AC080038.3 | 2.78612973 | 1.154473846 | -1.27E+00 | 0.0005245 | 0.0042142 |
| ADM | 2.841852703 | 6.178010769 | 1.12E+00 | 0.0017981 | 0.0096658 |
| MIR8057 | 5.439132432 | 2.606113846 | -1.06E+00 | 0.0085176 | 0.0286448 |
| MIR6515 | 2.356843243 | 0.732883077 | -1.69E+00 | 0.0005568 | 0.0043819 |
| TNFRSF1B | 94.10723649 | 194.9154077 | 1.05E+00 | 3.46E-06 | 0.0002269 |
| AC007877.1 | 1.780810811 | 5.396258462 | 1.60E+00 | 6.29E-08 | 3.29E-05 |
| HLA-F | 16.73631892 | 34.78617692 | 1.06E+00 | 2.30E-09 | 1.05E-05 |
| MIR4319 | 3.316359459 | 8.803335385 | 1.41E+00 | 0.0030507 | 0.0139237 |
| ABI3 | 11.21903514 | 26.46246923 | 1.24E+00 | 1.36E-05 | 0.0004554 |
| LTK | 34.95294865 | 15.36256923 | -1.19E+00 | 0.0035585 | 0.0154661 |
| TPRG1-AS1 | 0.843691892 | 2.351121538 | 1.48E+00 | 0.0001548 | 0.0019272 |
| FHL2 | 1.398006757 | 5.092427692 | 1.86E+00 | 0.0092552 | 0.0303321 |
| LILRB4 | 12.5858973 | 27.22428308 | 1.11E+00 | 1.50E-05 | 0.0004778 |
| ZNF385C | 9.479205405 | 3.410641538 | -1.47E+00 | 7.71E-05 | 0.0012374 |
| AC073850.1 | 18.36768108 | 42.03832154 | 1.19E+00 | 0.0048971 | 0.0193958 |
| SERPINE1 | 19.93566622 | 7.698673846 | -1.37E+00 | 0.0026321 | 0.0125425 |
| CPAMD8 | 0.7257 | 2.144121538 | 1.56E+00 | 0.0011608 | 0.0070965 |
| AC093591.2 | 1.741213514 | 0.708281538 | -1.30E+00 | 1.57E-05 | 0.000497 |
| MPO | 8887.992785 | 3164.1244 | -1.49E+00 | 0.0005499 | 0.0043306 |
| ZNF33BP1 | 1.772327027 | 0.851847692 | -1.06E+00 | 2.54E-06 | 0.0001965 |
| AL645608.6 | 20.26765405 | 9.549564615 | -1.09E+00 | 0.0044937 | 0.0182637 |
| AL645608.2 | 15.24255676 | 6.691095385 | -1.19E+00 | 0.0011268 | 0.0069607 |
| CILP2 | 3.806635135 | 1.440852308 | -1.40E+00 | 0.0062694 | 0.022989 |
| HNMT | 6.827591892 | 16.61190308 | 1.28E+00 | 0.0053693 | 0.0206581 |
| LGALSL | 2.148247297 | 4.902692308 | 1.19E+00 | 0.0019313 | 0.0101279 |
| LILRB3 | 25.97423108 | 54.84995692 | 1.08E+00 | 7.88E-06 | 0.0003416 |
| AC104088.1 | 3.026902703 | 1.127426154 | -1.42E+00 | 0.014478 | 0.0418076 |
| CSF1 | 5.374187838 | 18.8697 | 1.81E+00 | 0.0003881 | 0.0034418 |
| SNORC | 2.456171622 | 1.183407692 | -1.05E+00 | 0.0014687 | 0.0083186 |
| AC007922.1 | 1.648891892 | 0.579555385 | -1.51E+00 | 0.0087121 | 0.0290559 |
| RNA5SP312 | 1.659508108 | 0.465861538 | -1.83E+00 | 7.31E-05 | 0.0012009 |
| AC060764.1 | 0.601733784 | 1.495629231 | 1.31E+00 | 0.0053345 | 0.0205981 |
| ECE1 | 10.93063649 | 22.42503846 | 1.04E+00 | 3.15E-07 | 6.47E-05 |
| LRRC25 | 35.24585135 | 83.30846615 | 1.24E+00 | 3.01E-05 | 0.0007191 |
| AC115621.1 | 1.69567027 | 0.783090769 | -1.11E+00 | 0.0007524 | 0.0052753 |
| HGF | 115.1785189 | 33.49214615 | -1.78E+00 | 0.0019039 | 0.0100218 |
| ITGB2-AS1 | 17.05950541 | 44.03131846 | 1.37E+00 | 2.88E-07 | 6.42E-05 |
| AC005197.1 | 1.653437838 | 0.7338 | -1.17E+00 | 2.31E-05 | 0.0006153 |
| AC027763.1 | 17.45633919 | 5.672066154 | -1.62E+00 | 0.0126015 | 0.0376957 |
| LINC02175 | 18.65667297 | 9.04176 | -1.05E+00 | 2.49E-06 | 0.0001965 |
| AGAP13P | 25.95204189 | 12.25116308 | -1.08E+00 | 0.0094857 | 0.0308222 |
| IL7 | 1.55915 | 3.3031 | 1.08E+00 | 0.0003819 | 0.0034059 |
| PDK4 | 8.395683784 | 27.00437846 | 1.69E+00 | 0.0008091 | 0.0055314 |
| AC073648.7 | 2.229145946 | 1.104193846 | -1.01E+00 | 3.12E-06 | 0.0002169 |
| CIAPIN1P | 3.862212162 | 1.569464615 | -1.30E+00 | 2.34E-06 | 0.0001921 |
| OR52K3P | 8.885831081 | 18.90208 | 1.09E+00 | 0.0016733 | 0.0091751 |
| NINJ1 | 22.9335473 | 52.19095077 | 1.19E+00 | 1.86E-06 | 0.000166 |
| HOXB5 | 7.126895946 | 15.72648154 | 1.14E+00 | 0.0011297 | 0.0069765 |
| TRDV2 | 18.47388784 | 9.031463077 | -1.03E+00 | 0.0001793 | 0.0020998 |
| SHISA4 | 0.97157027 | 2.457053846 | 1.34E+00 | 3.65E-05 | 0.0007919 |
| CXCR2P1 | 6.210722973 | 13.20461077 | 1.09E+00 | 0.0028013 | 0.0131183 |
| CASP5 | 0.911236486 | 3.766055385 | 2.05E+00 | 0.0003522 | 0.0032297 |
| ST18 | 7.474901351 | 2.544644615 | -1.55E+00 | 0.0027439 | 0.0129083 |
| AC098679.5 | 2.270966216 | 6.358852308 | 1.49E+00 | 1.89E-05 | 0.0005519 |
| MAFB | 31.92371216 | 75.30501385 | 1.24E+00 | 6.57E-05 | 0.0011273 |
| TRAV28 | 3.065433784 | 0.937232308 | -1.71E+00 | 0.0004115 | 0.0035934 |
| ITGAX | 56.85730541 | 125.9298492 | 1.15E+00 | 4.07E-06 | 0.0002436 |
| MIR199B | 2.811028378 | 1.22986 | -1.19E+00 | 0.0007966 | 0.0054794 |
| MX1 | 8.579971622 | 20.51988308 | 1.26E+00 | 4.04E-05 | 0.0008441 |
| SPATS2L | 1.805025676 | 3.667810769 | 1.02E+00 | 1.82E-06 | 0.0001652 |
| LINC02244 | 7.557163514 | 3.352926154 | -1.17E+00 | 2.27E-05 | 0.0006088 |
| AC009774.1 | 15.62662027 | 7.76646 | -1.01E+00 | 0.0001758 | 0.0020821 |
| SLC24A3-AS1 | 10.84260405 | 4.308141538 | -1.33E+00 | 0.000693 | 0.0050235 |
| SRGAP3-AS2 | 1.604 | 0.448487692 | -1.84E+00 | 3.16E-05 | 0.0007374 |
| LILRB1 | 14.03443784 | 38.43182923 | 1.45E+00 | 4.04E-05 | 0.0008441 |
| MIR651 | 2.650668919 | 1.314841538 | -1.01E+00 | 0.0022496 | 0.011299 |
| MAP1A | 42.83432568 | 20.10813692 | -1.09E+00 | 0.0120865 | 0.0365488 |
| ZNF703 | 2.73627027 | 8.253286154 | 1.59E+00 | 0.0019313 | 0.0101279 |
| ARPP21-AS1 | 6.500402703 | 2.606406154 | -1.32E+00 | 0.0006438 | 0.0047948 |
| AC099489.3 | 2.646490541 | 1.255629231 | -1.08E+00 | 2.29E-06 | 0.0001902 |
| MIR4537 | 10.40959324 | 23.30842154 | 1.16E+00 | 0.0036964 | 0.0158905 |
| CD4 | 64.0331027 | 149.2333123 | 1.22E+00 | 9.43E-07 | 0.0001128 |
| FP236383.4 | 7.978940541 | 3.958949231 | -1.01E+00 | 0.0014712 | 0.0083306 |
| ADAP2 | 10.49511216 | 22.91354615 | 1.13E+00 | 0.000212 | 0.0023479 |
| IL4I1 | 1.382428378 | 4.191850769 | 1.60E+00 | 3.19E-06 | 0.0002186 |
| MYCT1 | 2.086806757 | 15.48123231 | 2.89E+00 | 0.0004163 | 0.003615 |
| SMIM1 | 3.471713514 | 7.503493846 | 1.11E+00 | 0.0037056 | 0.0158905 |
| LINC02131 | 2.624705405 | 0.540692308 | -2.28E+00 | 2.93E-05 | 0.0007122 |
| GBP1P1 | 1.527291892 | 3.303055385 | 1.11E+00 | 0.0165917 | 0.0459924 |
| SIGLEC1 | 4.75817973 | 16.75616 | 1.82E+00 | 0.0005084 | 0.004122 |
| VNN1 | 23.35344595 | 47.25491385 | 1.02E+00 | 3.06E-05 | 0.0007256 |
| CMTM5 | 0.795789189 | 5.583430769 | 2.81E+00 | 1.67E-05 | 0.0005147 |
| AC105235.1 | 0.815033784 | 1.673426154 | 1.04E+00 | 3.26E-05 | 0.000751 |
| CD96 | 152.9009703 | 71.74193538 | -1.09E+00 | 0.0027061 | 0.0127682 |
| IGHE | 0.848904054 | 3.122455385 | 1.88E+00 | 1.81E-05 | 0.0005371 |
| LPAR4 | 13.42051216 | 6.013266154 | -1.16E+00 | 1.10E-05 | 0.0004035 |
| CD300H | 7.378321622 | 17.46625077 | 1.24E+00 | 0.0017807 | 0.0096164 |
| AC005336.1 | 5.744290541 | 0.907341538 | -2.66E+00 | 0.0038657 | 0.0164143 |
| GNGT2 | 1.644174324 | 3.431493846 | 1.06E+00 | 1.10E-05 | 0.0004035 |
| NCF1B | 11.13609459 | 25.42842 | 1.19E+00 | 9.51E-05 | 0.0014172 |
| NAMPTP1 | 2.336312162 | 5.569035385 | 1.25E+00 | 0.0009999 | 0.0063871 |
| AC104088.2 | 2.037837838 | 0.755321538 | -1.43E+00 | 0.0039323 | 0.0166312 |
| CHST15 | 13.90748514 | 27.89811385 | 1.00E+00 | 0.0005004 | 0.0040841 |
| AC007540.1 | 2.623464865 | 1.308930769 | -1.00E+00 | 0.0008165 | 0.0055802 |
| ANKRD33B-AS1 | 4.521093243 | 2.239044615 | -1.01E+00 | 2.49E-06 | 0.0001965 |
| SERPINA1 | 81.01799459 | 184.0139954 | 1.18E+00 | 0.0177735 | 0.0483271 |
| CDK6-AS1 | 57.43395405 | 18.31102923 | -1.65E+00 | 1.65E-05 | 0.0005092 |
| AC133552.4 | 36.45292432 | 17.61320308 | -1.05E+00 | 2.54E-06 | 0.0001965 |
| NCF1 | 15.70996757 | 38.54603385 | 1.29E+00 | 4.19E-05 | 0.0008533 |
| AC009237.8 | 0.682543243 | 1.572161538 | 1.20E+00 | 0.0041152 | 0.017141 |
| AC008659.1 | 9.957237838 | 3.874766154 | -1.36E+00 | 0.0062599 | 0.022989 |
| MIR181A1HG | 22.60337973 | 8.998913846 | -1.33E+00 | 0.0001234 | 0.0016645 |
| RNU6-1300P | 1.289094595 | 3.20442 | 1.31E+00 | 0.0025073 | 0.0121723 |
| PPATP2 | 2.125964865 | 0.7294 | -1.54E+00 | 6.77E-05 | 0.0011476 |
| TRAV40 | 1.559267568 | 0.678172308 | -1.20E+00 | 0.0025987 | 0.0124361 |
| EPSTI1 | 6.410271622 | 14.81048154 | 1.21E+00 | 2.27E-06 | 0.0001896 |
| SYT17 | 0.662268919 | 1.560283077 | 1.24E+00 | 0.0019871 | 0.0103289 |
| CPED1 | 1.460585135 | 4.383747692 | 1.59E+00 | 0.0002421 | 0.0025232 |
| STOX2 | 2.619387838 | 0.769152308 | -1.77E+00 | 0.0033706 | 0.0148506 |
| SPART-AS1 | 5.896714865 | 2.944181538 | -1.00E+00 | 2.80E-08 | 2.49E-05 |
| AC132807.2 | 44.88736486 | 10.82530154 | -2.05E+00 | 1.90E-06 | 0.0001675 |
| ISG15 | 20.15938378 | 41.16670462 | 1.03E+00 | 9.68E-05 | 0.0014288 |
| TRAV37 | 2.687035135 | 0.884001538 | -1.60E+00 | 0.0005328 | 0.004249 |
| ITM2A | 263.3578243 | 127.1199338 | -1.05E+00 | 0.0033251 | 0.0147308 |
| IFITM10 | 0.823395946 | 1.71376 | 1.06E+00 | 0.0156603 | 0.0441892 |
| AP001099.1 | 2.368917568 | 5.582744615 | 1.24E+00 | 0.0094083 | 0.0307264 |
| TRBV12-1 | 2.743325676 | 1.139938462 | -1.27E+00 | 0.0002694 | 0.0026933 |
| AC080037.1 | 44.40230135 | 21.51328462 | -1.05E+00 | 9.74E-07 | 0.0001153 |
| PPM1N | 2.309516216 | 5.263230769 | 1.19E+00 | 0.0002807 | 0.0027606 |
| MIR100HG | 1.983551351 | 0.289730769 | -2.78E+00 | 0.0007049 | 0.0050672 |
| CERCAM | 28.66651351 | 13.20239538 | -1.12E+00 | 0.0156603 | 0.0441892 |
| DPP4 | 0.738601351 | 1.70294 | 1.21E+00 | 0.0007848 | 0.0054179 |
| SRGAP3-AS3 | 1.804686486 | 0.626610769 | -1.53E+00 | 0.000309 | 0.0029478 |
| RNU7-134P | 10.37883649 | 5.147010769 | -1.01E+00 | 0.0006854 | 0.0049909 |
| KIR2DL4 | 0.507428378 | 1.830152308 | 1.85E+00 | 4.38E-05 | 0.0008788 |
| RPL37P18 | 1.383041892 | 0.631964615 | -1.13E+00 | 0.0182707 | 0.0493568 |
| SRGAP3 | 4.351612162 | 1.691912308 | -1.36E+00 | 0.0028996 | 0.013403 |
| FOLR2 | 1.863010811 | 6.698315385 | 1.85E+00 | 7.51E-05 | 0.0012207 |
| AC093520.2 | 4.615652703 | 9.262915385 | 1.00E+00 | 0.0005155 | 0.0041708 |
| FP671120.6 | 16.53604189 | 5.050812308 | -1.71E+00 | 0.0071189 | 0.0251678 |
| AC244502.1 | 16.09164865 | 7.549667692 | -1.09E+00 | 0.0001299 | 0.0017266 |
| DHRS9 | 10.76701216 | 22.87127692 | 1.09E+00 | 0.0052312 | 0.0202688 |
| IL1RN | 10.77292838 | 23.93475385 | 1.15E+00 | 2.19E-05 | 0.0006014 |
| FCGR3A | 30.03751486 | 124.8964692 | 2.06E+00 | 0.0044089 | 0.0179941 |
| NBAT1 | 3.379854054 | 1.565475385 | -1.11E+00 | 0.0002172 | 0.0023801 |
| GP5 | 0.73682027 | 1.743449231 | 1.24E+00 | 0.0071211 | 0.0251678 |
| DUSP8 | 0.577422973 | 1.656964615 | 1.52E+00 | 1.80E-05 | 0.0005371 |
| AC245884.2 | 2.316991892 | 5.47606 | 1.24E+00 | 0.0002925 | 0.0028482 |
| SIX3 | 13.02895541 | 0.235270769 | -5.79E+00 | 0.0007803 | 0.0054169 |
| MIR151A | 1.39512027 | 2.888955385 | 1.05E+00 | 0.0026016 | 0.0124472 |
| FBLN2 | 2.449898649 | 6.307129231 | 1.36E+00 | 0.0139343 | 0.0406341 |
| AL118508.2 | 0.481951351 | 1.664333846 | 1.79E+00 | 5.49E-05 | 0.0010163 |
| ZSCAN23 | 2.726278378 | 1.220126154 | -1.16E+00 | 0.0011504 | 0.0070697 |
| KCNK1 | 0.100297297 | 3.497755385 | 5.12E+00 | 0.0045247 | 0.018344 |
| CTSL | 3.809033784 | 11.62665538 | 1.61E+00 | 2.23E-05 | 0.0006047 |
| DNM1 | 35.28756081 | 16.71022308 | -1.08E+00 | 0.0002996 | 0.0028789 |
| RPS6KA2-IT1 | 3.177760811 | 1.483372308 | -1.10E+00 | 0.0037341 | 0.0159947 |
| VANGL2 | 5.249474324 | 2.417610769 | -1.12E+00 | 0.0013453 | 0.0078655 |
| AJ009632.2 | 1.035167568 | 6.435401538 | 2.64E+00 | 0.0001047 | 0.0014941 |
| OSTCP6 | 2.035158108 | 0.659550769 | -1.63E+00 | 6.72E-05 | 0.0011406 |
| IFITM3 | 85.47790135 | 220.0428615 | 1.36E+00 | 1.96E-05 | 0.0005639 |
| MIR4507 | 5.772418919 | 12.33866 | 1.10E+00 | 0.005683 | 0.0215009 |
| PCBP3 | 7.695121622 | 2.465290769 | -1.64E+00 | 0.0163072 | 0.0454952 |
| TMSB4XP3 | 2.056277027 | 0.928392308 | -1.15E+00 | 0.0126307 | 0.0377752 |
| ZRANB2-AS2 | 1.659013514 | 0.692363077 | -1.26E+00 | 0.0004515 | 0.0038441 |
| RNU11-3P | 2.562539189 | 1.104536923 | -1.21E+00 | 1.64E-08 | 2.05E-05 |
| AC138649.2 | 1.366062162 | 4.089935385 | 1.58E+00 | 0.0113224 | 0.0349141 |
| NDST3 | 4.978502703 | 0.856 | -2.54E+00 | 0.0002711 | 0.0027047 |
| SEMA3C | 0.777371622 | 3.026210769 | 1.96E+00 | 0.0002994 | 0.0028789 |
| MRC2 | 31.99740541 | 15.44754462 | -1.05E+00 | 0.0069429 | 0.0247049 |
| AC020917.1 | 11.89602162 | 5.604161538 | -1.09E+00 | 0.0031918 | 0.0143267 |
| AC018645.1 | 2.718632432 | 1.041827692 | -1.38E+00 | 0.0003247 | 0.0030472 |
| LINC00482 | 4.827466216 | 12.99418154 | 1.43E+00 | 0.0022883 | 0.0114021 |
| NRGN | 65.36095 | 137.6248908 | 1.07E+00 | 5.02E-07 | 8.22E-05 |
| AC104071.1 | 0.333343243 | 3.865466154 | 3.54E+00 | 0.0121586 | 0.0367527 |
| AC010247.2 | 3.891021622 | 8.226726154 | 1.08E+00 | 4.93E-05 | 0.0009464 |
| RNASE1 | 6.287354054 | 24.80068308 | 1.98E+00 | 0.0022888 | 0.0114021 |
| HNRNPA1P62 | 7.30147973 | 3.310803077 | -1.14E+00 | 1.70E-05 | 0.0005189 |
| ZMYND15 | 0.786236486 | 1.868869231 | 1.25E+00 | 0.0094856 | 0.0308222 |
| LILRA6 | 18.77601622 | 40.59806154 | 1.11E+00 | 5.70E-05 | 0.001044 |
| EFCC1 | 6.584560811 | 15.23594462 | 1.21E+00 | 0.0068118 | 0.0244202 |
| TIMP3 | 6.962131081 | 24.06204615 | 1.79E+00 | 0.0151238 | 0.0430641 |
| RDM1P3 | 12.71924459 | 6.237916923 | -1.03E+00 | 6.73E-06 | 0.000312 |
| AC064805.1 | 3.15837027 | 8.155235385 | 1.37E+00 | 9.68E-05 | 0.0014288 |
| HLF | 1.033928378 | 4.271461538 | 2.05E+00 | 0.0011781 | 0.0071674 |
| BEX5 | 0.727037838 | 1.767141538 | 1.28E+00 | 0.0041061 | 0.0171282 |
| BMS1P2 | 5.171777027 | 1.851269231 | -1.48E+00 | 2.32E-05 | 0.0006153 |
| AL645608.4 | 1.863083784 | 0.710392308 | -1.39E+00 | 0.0037439 | 0.0160326 |
| MTND5P32 | 1.3744 | 3.942995385 | 1.52E+00 | 0.0117144 | 0.035799 |
| LINC01114 | 3.524495946 | 0.944061538 | -1.90E+00 | 0.0048037 | 0.019145 |
| ATG12P1 | 7.778668919 | 3.8861 | -1.00E+00 | 2.54E-06 | 0.0001965 |
| IL10 | 0.688632432 | 2.172306154 | 1.66E+00 | 0.0023209 | 0.0115124 |
| AC080037.2 | 5.447487838 | 2.148484615 | -1.34E+00 | 1.82E-06 | 0.0001652 |
| AL031985.1 | 3.852364865 | 7.950652308 | 1.05E+00 | 0.0155953 | 0.0440831 |
| AC132807.1 | 4.193314865 | 1.184010769 | -1.82E+00 | 6.13E-07 | 9.26E-05 |
| GIMAP4 | 30.18063378 | 63.80342154 | 1.08E+00 | 0.0054396 | 0.0208465 |
| CAVIN2 | 12.95676216 | 31.67356923 | 1.29E+00 | 0.0006835 | 0.0049803 |
| USP6 | 2.692086486 | 1.198373846 | -1.17E+00 | 0.0097807 | 0.0315532 |
| MST1R | 0.774922973 | 2.193766154 | 1.50E+00 | 0.0069869 | 0.0248501 |
| TBC1D12 | 1.426193243 | 3.101690769 | 1.12E+00 | 5.49E-05 | 0.0010163 |
| PID1 | 1.676955405 | 3.532467692 | 1.07E+00 | 0.0095412 | 0.0309984 |
| AC021766.1 | 2.433651351 | 5.067776923 | 1.06E+00 | 0.0135582 | 0.0398628 |
| AC073648.4 | 1.492218919 | 0.6982 | -1.10E+00 | 1.12E-05 | 0.0004055 |
| ZNF114 | 3.400136486 | 1.594564615 | -1.09E+00 | 1.01E-07 | 4.53E-05 |
| TMEM105 | 2.14222973 | 5.2243 | 1.29E+00 | 0.0151233 | 0.0430641 |
| AC245884.12 | 9.429481081 | 36.01531077 | 1.93E+00 | 0.0057778 | 0.0217569 |
| HHIP-AS1 | 3.843056757 | 0.915055385 | -2.07E+00 | 9.76E-05 | 0.0014385 |
| AL391903.3 | 1.785105405 | 3.838270769 | 1.10E+00 | 0.0002304 | 0.002455 |
| Z98745.2 | 6.696097297 | 2.471341538 | -1.44E+00 | 0.0001003 | 0.0014561 |
| ADGRA2 | 14.68333649 | 32.47308154 | 1.15E+00 | 0.000448 | 0.0038167 |
| TNNI2 | 4.98347027 | 12.88907846 | 1.37E+00 | 1.01E-05 | 0.0003887 |
| KCTD17 | 4.662201351 | 9.383709231 | 1.01E+00 | 1.96E-07 | 6.00E-05 |
| RPS2P4 | 15.51136351 | 6.475610769 | -1.26E+00 | 0.0040705 | 0.0169827 |
| JPH4 | 1.575954054 | 3.428835385 | 1.12E+00 | 0.0001918 | 0.0021931 |
| RPL32P33 | 1.525954054 | 0.726476923 | -1.07E+00 | 9.08E-05 | 0.0013856 |
| AC126544.1 | 40.4201973 | 19.45233846 | -1.06E+00 | 0.0001299 | 0.0017266 |
| LINC02147 | 5.036635135 | 2.406744615 | -1.07E+00 | 0.0015409 | 0.0086386 |
| APOBEC3A | 12.56119595 | 31.56342154 | 1.33E+00 | 0.0001734 | 0.0020614 |
| CCR5AS | 3.055766216 | 6.524921538 | 1.09E+00 | 5.90E-05 | 0.0010648 |
| PPBP | 34.62659189 | 113.6365446 | 1.71E+00 | 0.0001391 | 0.0018051 |
| AC099508.2 | 1.807972973 | 0.759567692 | -1.25E+00 | 0.0081789 | 0.0277675 |
| CRNDE | 14.30967027 | 4.325998462 | -1.73E+00 | 2.69E-05 | 0.0006747 |
| CX3CR1 | 54.99019865 | 140.2671662 | 1.35E+00 | 0.0001234 | 0.0016645 |
| CXCL16 | 8.695017568 | 21.38295538 | 1.30E+00 | 0.0016257 | 0.008973 |
| TRGJP | 16.70077027 | 8.302793846 | -1.01E+00 | 0.0008598 | 0.0057678 |
| AC099489.1 | 23.15304459 | 7.863941538 | -1.56E+00 | 0.0037056 | 0.0158905 |
| ARPP21 | 4.149454054 | 1.770604615 | -1.23E+00 | 0.0008248 | 0.0056129 |
| AC112482.2 | 4.728747297 | 2.082167692 | -1.18E+00 | 0.0030043 | 0.0137764 |
| KCNQ1 | 7.144701351 | 15.84280308 | 1.15E+00 | 5.85E-07 | 9.14E-05 |
| FOLR3 | 2.625378378 | 5.765527692 | 1.13E+00 | 0.0014121 | 0.0081398 |
| LINC01833 | 4.310401351 | 0.142446154 | -4.92E+00 | 7.45E-05 | 0.0012132 |
| COL2A1 | 4.903751351 | 0.595127692 | -3.04E+00 | 9.94E-07 | 0.0001158 |
| CXCR1 | 1.74447973 | 4.061729231 | 1.22E+00 | 0.0004811 | 0.0039986 |
| MIR4799 | 1.658272973 | 0.663081538 | -1.32E+00 | 0.0026982 | 0.0127682 |
| FCN1 | 87.87541081 | 177.8852123 | 1.02E+00 | 0.0003198 | 0.0030145 |
| RFX8 | 76.46315 | 35.42284 | -1.11E+00 | 0.0006039 | 0.0046151 |
| SMPDL3A | 3.398621622 | 7.121090769 | 1.07E+00 | 0.0030633 | 0.013934 |
| SHD | 14.94406081 | 5.92884 | -1.33E+00 | 0.0039622 | 0.0166985 |
| HFE | 0.888021622 | 2.018541538 | 1.18E+00 | 0.0016257 | 0.008973 |
| RNU2-59P | 2.803166216 | 1.271856923 | -1.14E+00 | 9.67E-05 | 0.0014288 |
| KIF26B-AS1 | 5.045286486 | 1.320316923 | -1.93E+00 | 0.0001208 | 0.001645 |
| MIR4538 | 5.636489189 | 12.57707846 | 1.16E+00 | 0.0025869 | 0.0124246 |
| IGHM | 334.0371757 | 713.0720538 | 1.09E+00 | 7.44E-05 | 0.0012117 |
| AFF2-IT1 | 4.421462162 | 1.25904 | -1.81E+00 | 0.0001145 | 0.0015856 |
| AC018645.2 | 1.756952703 | 0.649584615 | -1.44E+00 | 2.91E-05 | 0.0007081 |
| GASK1B | 12.70863243 | 27.12349538 | 1.09E+00 | 0.0013853 | 0.0080347 |
| JAKMIP2 | 0.993418919 | 2.436983077 | 1.29E+00 | 0.0005586 | 0.0043866 |
| RN7SL646P | 16.42313784 | 5.981387692 | -1.46E+00 | 0.0011738 | 0.0071674 |
| ODF3B | 16.81874054 | 38.54603077 | 1.20E+00 | 0.0003943 | 0.0034768 |
| GPR25 | 0.45972027 | 2.977153846 | 2.70E+00 | 0.0004068 | 0.0035592 |
| AC005336.3 | 22.78334595 | 6.991704615 | -1.70E+00 | 0.005029 | 0.019744 |
| GLB1L2 | 1.046967568 | 2.144129231 | 1.03E+00 | 0.0009703 | 0.0062478 |
| GPBAR1 | 6.901060811 | 15.42241538 | 1.16E+00 | 0.0014687 | 0.0083186 |
| AC083855.2 | 3.321791892 | 6.855244615 | 1.05E+00 | 0.0011084 | 0.0068881 |
| IER3 | 3.353895946 | 8.524863077 | 1.35E+00 | 1.65E-05 | 0.0005092 |
| AL627309.6 | 6.254290541 | 16.72555231 | 1.42E+00 | 7.05E-05 | 0.0011754 |
| USP41 | 3.671713514 | 1.820647692 | -1.01E+00 | 0.0028996 | 0.013403 |
| EPHX2 | 1.074544595 | 3.281106154 | 1.61E+00 | 5.59E-05 | 0.0010322 |
| SLC24A3 | 16.41643784 | 7.164675385 | -1.20E+00 | 0.0085896 | 0.0287763 |
| AC139792.2 | 2.814933784 | 1.405870769 | -1.00E+00 | 3.12E-06 | 0.0002169 |
| NT5C3AP2 | 72.85943108 | 31.30321385 | -1.22E+00 | 0.0002342 | 0.0024814 |
| CPNE8-AS1 | 2.054794595 | 4.287215385 | 1.06E+00 | 9.84E-05 | 0.0014416 |
| RPS20P22 | 3.77692027 | 1.326829231 | -1.51E+00 | 0.0005246 | 0.0042142 |
| PACSIN1 | 3.420536486 | 8.36904 | 1.29E+00 | 0.006987 | 0.0248501 |
| CYP2E1 | 2.409347297 | 1.005533846 | -1.26E+00 | 0.0014687 | 0.0083186 |
| CLEC4O | 158.1005365 | 66.94850154 | -1.24E+00 | 0.0047608 | 0.0190399 |
| AL136090.1 | 4.951072973 | 2.336793846 | -1.08E+00 | 0.0099895 | 0.0320244 |
| NTNG2 | 76.88207432 | 27.11986308 | -1.50E+00 | 4.50E-05 | 0.000891 |
| HSPA4L | 3.076616216 | 1.210433846 | -1.35E+00 | 0.000154 | 0.0019184 |
| MTUS2 | 2.544371622 | 0.636587692 | -2.00E+00 | 0.0007269 | 0.0051562 |
| PELATON | 5.844054054 | 15.33501692 | 1.39E+00 | 0.0002421 | 0.0025232 |
| GBP1 | 5.975808108 | 17.83943077 | 1.58E+00 | 0.0006327 | 0.0047371 |
| IFI30 | 2.771352703 | 7.516281538 | 1.44E+00 | 4.27E-05 | 0.0008604 |
| SNORA68B | 29.79144324 | 14.51094 | -1.04E+00 | 4.11E-10 | 4.62E-06 |
| AL359091.4 | 3.278010811 | 1.522496923 | -1.11E+00 | 0.0003045 | 0.002915 |
| OASL | 2.529613514 | 6.214878462 | 1.30E+00 | 6.09E-06 | 0.0002946 |
| SIGLEC11 | 0.835704054 | 1.901538462 | 1.19E+00 | 0.0093523 | 0.030605 |
| AL357033.2 | 1.89992973 | 0.946324615 | -1.01E+00 | 0.0043782 | 0.0179238 |
| PLA2G4C | 1.028332432 | 2.076712308 | 1.01E+00 | 0.0010937 | 0.0068069 |
| NCF1C | 19.02340811 | 42.62515538 | 1.16E+00 | 7.18E-05 | 0.0011885 |
| TRAV33 | 2.632181081 | 1.092936923 | -1.27E+00 | 0.0060015 | 0.0223339 |
| ALDH1A1 | 9.383967568 | 32.76882923 | 1.80E+00 | 0.0007049 | 0.0050672 |
| SLC15A3 | 6.520806757 | 14.56246462 | 1.16E+00 | 8.53E-06 | 0.0003559 |
| SASH1 | 0.597410811 | 2.100149231 | 1.81E+00 | 0.0168804 | 0.0465916 |
| FLNC | 1.620294595 | 0.403621538 | -2.01E+00 | 0.001973 | 0.0102889 |
| AL022316.1 | 1.380252703 | 3.234887692 | 1.23E+00 | 0.0002846 | 0.0027914 |
| LY6G6E | 0.54637027 | 1.60876 | 1.56E+00 | 3.51E-05 | 0.0007772 |
| HRH4 | 2.178235135 | 0.932701538 | -1.22E+00 | 0.0006426 | 0.0047872 |
| ITGB3 | 6.634983784 | 17.70377231 | 1.42E+00 | 0.0001734 | 0.0020614 |
| AC009075.2 | 2.305791892 | 1.122556923 | -1.04E+00 | 1.17E-07 | 4.97E-05 |
| RNU6-446P | 7.956814865 | 2.632641538 | -1.60E+00 | 0.0003308 | 0.0030795 |
| AC136601.1 | 9.536601351 | 3.637635385 | -1.39E+00 | 0.0022161 | 0.0111659 |
| ADGRG6 | 1.987563514 | 14.00535231 | 2.82E+00 | 0.0086462 | 0.0289421 |
| UBXN10 | 0.737917568 | 2.124003077 | 1.53E+00 | 0.0138523 | 0.0404789 |
| GNAZ | 0.735698649 | 1.707990769 | 1.22E+00 | 3.55E-05 | 0.0007772 |
| HK3 | 40.27564324 | 111.79426 | 1.47E+00 | 5.19E-06 | 0.0002746 |
| TRBV12-2 | 4.502359459 | 2.241473846 | -1.01E+00 | 0.0009477 | 0.0061625 |
| MIR181A1 | 3.333539189 | 1.175863077 | -1.50E+00 | 9.07E-07 | 0.0001128 |
| LILRA1 | 24.82376351 | 51.98382923 | 1.07E+00 | 7.44E-05 | 0.0012117 |
| AC022726.2 | 17.11966081 | 8.530113846 | -1.01E+00 | 6.67E-07 | 9.44E-05 |
| PDZK1IP1 | 1.576375676 | 4.425343077 | 1.49E+00 | 0.0010853 | 0.0067733 |
| AL844908.1 | 2.938893243 | 5.965472308 | 1.02E+00 | 6.15E-06 | 0.0002969 |
| THCAT158 | 1.094287838 | 2.576329231 | 1.24E+00 | 0.0062246 | 0.0228987 |
| RN7SKP267 | 1.69592973 | 0.513533846 | -1.72E+00 | 0.0026732 | 0.0126822 |
| RRAS | 22.98823514 | 46.98698615 | 1.03E+00 | 6.60E-06 | 0.0003084 |
| TRAV34 | 3.316574324 | 1.229221538 | -1.43E+00 | 0.0024762 | 0.0120788 |
| SMC5-AS1 | 10.3748473 | 4.395281538 | -1.24E+00 | 0.0006942 | 0.0050235 |
| MTATP8P1 | 83.81512432 | 18.88451692 | -2.15E+00 | 0.014099 | 0.040971 |
| TGM2 | 2.737531081 | 8.154958462 | 1.57E+00 | 0.0055827 | 0.0212106 |
| ABCC3 | 1.431012162 | 4.448656923 | 1.64E+00 | 6.23E-05 | 0.0010979 |
| METTL7B | 11.99341892 | 35.96482923 | 1.58E+00 | 0.0001299 | 0.0017266 |
| KRT17P8 | 1.601193243 | 3.226916923 | 1.01E+00 | 0.0048993 | 0.0193958 |
| THRAP3P3 | 4.064785135 | 0.665386154 | -2.61E+00 | 0.002443 | 0.0119581 |
| FAM198B-AS1 | 2.128512162 | 5.457286154 | 1.36E+00 | 1.06E-05 | 0.0003992 |
| NDFIP2 | 5.032985135 | 2.04582 | -1.30E+00 | 0.0055108 | 0.0210224 |
| AC064834.1 | 2.560613514 | 1.101972308 | -1.22E+00 | 0.0006928 | 0.0050235 |
| LINC00677 | 1.175137838 | 2.550681538 | 1.12E+00 | 0.0011866 | 0.0072173 |
| MARCO | 4.313845946 | 18.52897385 | 2.10E+00 | 0.0001592 | 0.0019543 |
| NEURL1 | 2.464728378 | 6.671615385 | 1.44E+00 | 3.75E-05 | 0.0008032 |
| CALCRL | 6.19947973 | 13.38489692 | 1.11E+00 | 0.0034167 | 0.0149891 |
| TRAV31 | 1.77842973 | 0.605172308 | -1.56E+00 | 0.0010173 | 0.0064527 |
| AL357315.1 | 2.450286486 | 1.166675385 | -1.07E+00 | 0.0004779 | 0.0039736 |
| MMP2 | 49.94836486 | 20.20035077 | -1.31E+00 | 0.008919 | 0.0295531 |
| AC004381.3 | 57.05047297 | 24.33461385 | -1.23E+00 | 0.0013064 | 0.0077201 |
| IGHJ2P | 22.75866351 | 69.41210615 | 1.61E+00 | 0.0054614 | 0.0209265 |
| TPTEP1 | 3.446182432 | 1.21532 | -1.50E+00 | 0.0051633 | 0.020082 |
| ABCA9 | 1.109456757 | 2.452349231 | 1.14E+00 | 0.0005413 | 0.0042903 |
| NECTIN1-AS1 | 2.443532432 | 0.636792308 | -1.94E+00 | 0.0001185 | 0.0016257 |
| LINC01506 | 2.352910811 | 4.955278462 | 1.07E+00 | 0.000215 | 0.0023629 |
| C3orf56 | 1.698293243 | 0.515427692 | -1.72E+00 | 0.0004394 | 0.0037732 |
| SRPX2 | 0.672017568 | 1.777563077 | 1.40E+00 | 0.0022238 | 0.0111848 |
| LINC00707 | 6.101177027 | 1.464775385 | -2.06E+00 | 0.0072574 | 0.025565 |
| AL136090.2 | 5.322778378 | 2.282572308 | -1.22E+00 | 0.0102125 | 0.0324436 |
| BEND6 | 4.402332432 | 1.490093846 | -1.56E+00 | 0.0050625 | 0.0198496 |
| AC245884.11 | 1.569982432 | 3.518816923 | 1.16E+00 | 0.0026996 | 0.0127682 |
| VASH2 | 1.669152703 | 0.748444615 | -1.16E+00 | 0.0004305 | 0.0037115 |
| ITLN1 | 0.623728378 | 1.549449231 | 1.31E+00 | 0.0061901 | 0.0227833 |
| LINC01222 | 2.000072973 | 0.360067692 | -2.47E+00 | 8.31E-05 | 0.0013051 |
| HMGA2-AS1 | 2.882248649 | 7.898624615 | 1.45E+00 | 0.0005628 | 0.0044148 |
| RNA5SP207 | 2.180354054 | 5.702396923 | 1.39E+00 | 0.0003479 | 0.0032113 |
| H1-2 | 30.80577703 | 86.20882769 | 1.48E+00 | 7.98E-05 | 0.0012664 |
| TRGJ1 | 10.88625405 | 5.302770769 | -1.04E+00 | 0.0031154 | 0.0141019 |
| AC116351.1 | 6.747128378 | 1.1897 | -2.50E+00 | 8.39E-05 | 0.0013119 |
| MYOF | 10.25664595 | 23.11976462 | 1.17E+00 | 0.0005899 | 0.0045456 |
| AC021915.2 | 2.906255405 | 0.690127692 | -2.07E+00 | 1.10E-05 | 0.0004035 |
| IGFBP2 | 44.29475676 | 20.06312154 | -1.14E+00 | 0.0099619 | 0.0319404 |
| KIR3DL1 | 0.486274324 | 1.642156923 | 1.76E+00 | 4.50E-06 | 0.000256 |
| G0S2 | 8.581156757 | 19.55474615 | 1.19E+00 | 0.0085936 | 0.0287763 |
| FCGR2C | 9.797986486 | 28.72329385 | 1.55E+00 | 0.0083828 | 0.0282805 |
| CORO1CP1 | 5.802212162 | 2.578827692 | -1.17E+00 | 0.0008911 | 0.0059104 |
| ESPN | 0.956432432 | 2.299895385 | 1.27E+00 | 0.01361 | 0.0398901 |
| AL627309.7 | 30.26850541 | 72.26661538 | 1.26E+00 | 0.0002051 | 0.0022958 |
| AL392086.3 | 4.561293243 | 1.214347692 | -1.91E+00 | 0.0037056 | 0.0158905 |
| DPY19L2 | 25.56380541 | 11.49830154 | -1.15E+00 | 2.32E-05 | 0.0006153 |
| H2AC9P | 3.804940541 | 9.164544615 | 1.27E+00 | 0.0039323 | 0.0166312 |
| SCO2 | 1.125264865 | 2.680255385 | 1.25E+00 | 2.52E-05 | 0.0006509 |
| CD300LB | 14.75153243 | 30.53098462 | 1.05E+00 | 0.000195 | 0.00222 |
| LILRA5 | 18.71379324 | 44.20024923 | 1.24E+00 | 5.02E-05 | 0.0009571 |
| BMS1P1 | 31.82117297 | 14.10370615 | -1.17E+00 | 0.0004137 | 0.0035941 |
| RFLNA | 2.227059459 | 0.905133846 | -1.30E+00 | 0.0016493 | 0.009068 |
| RNU2-5P | 12.16431081 | 3.726029231 | -1.71E+00 | 4.88E-06 | 0.0002635 |
| OGDHL | 1.695556757 | 0.235286154 | -2.85E+00 | 0.0117892 | 0.0359501 |
| KCNC3 | 2.351364865 | 5.228563077 | 1.15E+00 | 8.34E-05 | 0.0013086 |
| MIR7152 | 5.694371622 | 13.36041538 | 1.23E+00 | 0.0001042 | 0.0014903 |
| AP001485.1 | 2.430363514 | 0.638243077 | -1.93E+00 | 0.0033071 | 0.0147033 |
| AC105150.1 | 4.988418919 | 2.26584 | -1.14E+00 | 0.0021178 | 0.0108195 |
| AL121830.1 | 1.482404054 | 0.529670769 | -1.48E+00 | 0.0010907 | 0.0068047 |
| MSR1 | 2.826104054 | 6.913563077 | 1.29E+00 | 0.002404 | 0.0118161 |
| RNU6-697P | 1.540278378 | 0.718586154 | -1.10E+00 | 0.0012867 | 0.0076294 |
| AGMO | 1.744393243 | 0.440070769 | -1.99E+00 | 0.0008393 | 0.0056759 |
| AC069277.1 | 1.543041892 | 0.54474 | -1.50E+00 | 0.0014656 | 0.0083186 |
| RS1 | 3.368904054 | 0.139638462 | -4.59E+00 | 0.00605 | 0.0224216 |
| IL15 | 1.628691892 | 3.940610769 | 1.27E+00 | 3.12E-05 | 0.0007314 |
| DNAJC19P8 | 3.122691892 | 0.964647692 | -1.69E+00 | 0.0001201 | 0.0016372 |
| MIR4539 | 16.65981351 | 45.02887385 | 1.43E+00 | 0.0002453 | 0.0025475 |
| PF4 | 16.21223243 | 56.33037538 | 1.80E+00 | 5.35E-06 | 0.000279 |
| RNA5SP354 | 17.98322432 | 8.818146154 | -1.03E+00 | 3.53E-06 | 0.0002276 |
| LINC01645 | 4.676608108 | 0.918161538 | -2.35E+00 | 0.0161679 | 0.0452505 |
| IFITM3P1 | 0.825567568 | 1.657033846 | 1.01E+00 | 0.0010586 | 0.006651 |
| AL161645.1 | 1.861574324 | 0.691430769 | -1.43E+00 | 2.73E-06 | 0.0002021 |
| PDCL3P5 | 3.037578378 | 1.003764615 | -1.60E+00 | 0.0003195 | 0.0030145 |
| RNA5SP165 | 3.954187838 | 1.422747692 | -1.47E+00 | 1.97E-07 | 6.00E-05 |
| AC136601.2 | 8.265708108 | 2.800192308 | -1.56E+00 | 0.0066943 | 0.024091 |
| AC037479.1 | 3.004386486 | 0.994375385 | -1.60E+00 | 0.0091066 | 0.0300507 |
| S100A16 | 6.578216216 | 19.66977231 | 1.58E+00 | 0.0113891 | 0.0350238 |
| LRRC28 | 18.22194324 | 9.060384615 | -1.01E+00 | 0.0004072 | 0.0035592 |
| H2AW | 3.452397297 | 7.560730769 | 1.13E+00 | 6.34E-06 | 0.0003027 |
| BCL3 | 29.54090135 | 60.60762769 | 1.04E+00 | 3.52E-07 | 6.66E-05 |
| THSD7A | 8.530351351 | 4.220883077 | -1.02E+00 | 0.0165912 | 0.0459924 |
| AC022726.1 | 14.31364865 | 6.752092308 | -1.08E+00 | 2.55E-08 | 2.49E-05 |
| AC004381.4 | 25.33554459 | 10.00689385 | -1.34E+00 | 0.0006229 | 0.0047049 |
| AC006504.6 | 1.601474324 | 0.556075385 | -1.53E+00 | 0.0004738 | 0.0039629 |
| RNA5SP311 | 45.02001486 | 18.09759846 | -1.31E+00 | 0.0007159 | 0.0051163 |
| MARK3P1 | 3.607922973 | 1.729009231 | -1.06E+00 | 0.000242 | 0.0025232 |
| SLC8A1 | 1.813555405 | 4.428683077 | 1.29E+00 | 4.98E-06 | 0.0002657 |
| GLUD1P2 | 7.553474324 | 2.507927692 | -1.59E+00 | 0.0029395 | 0.0135276 |
| C5AR1 | 48.75772027 | 113.1765169 | 1.21E+00 | 0.0003819 | 0.0034059 |
| PLXNB1 | 13.01803378 | 6.36776 | -1.03E+00 | 0.0022569 | 0.011303 |
| RN7SL138P | 10.47244189 | 22.88846 | 1.13E+00 | 0.0006133 | 0.0046558 |
| PLCH1 | 5.356408108 | 1.353983077 | -1.98E+00 | 0.0003495 | 0.0032221 |
| MTND6P4 | 60.77862838 | 23.09550923 | -1.40E+00 | 0.0007496 | 0.0052572 |
| ZBED2 | 16.55719054 | 7.494855385 | -1.14E+00 | 0.0008341 | 0.0056424 |
| NNMT | 4.353925676 | 1.918295385 | -1.18E+00 | 0.0023212 | 0.0115124 |
| IGHJ1 | 14.12432703 | 32.18990769 | 1.19E+00 | 0.0057907 | 0.0217744 |
| MAOB | 0.267882432 | 3.124158462 | 3.54E+00 | 0.0147937 | 0.0423764 |
| LAG3 | 2.520559459 | 6.122615385 | 1.28E+00 | 0.0038066 | 0.0162066 |
| SDC3 | 2.451154054 | 6.65328 | 1.44E+00 | 0.0098409 | 0.0316472 |
| AJ011931.1 | 1.587666216 | 0.418873846 | -1.92E+00 | 0.0028193 | 0.0131601 |
| TTC22 | 0.804293243 | 1.678304615 | 1.06E+00 | 4.84E-05 | 0.0009342 |
| AC096631.1 | 2.147758108 | 0.500883077 | -2.10E+00 | 0.0022712 | 0.0113698 |
| SLC28A3 | 12.17541351 | 2.439347692 | -2.32E+00 | 0.0005853 | 0.0045135 |
| PEAR1 | 6.212237838 | 21.45694154 | 1.79E+00 | 0.0011957 | 0.0072548 |
| SH3BP5 | 2.33732973 | 5.252238462 | 1.17E+00 | 8.70E-06 | 0.0003603 |
| AP002954.1 | 0.750708108 | 2.845764615 | 1.92E+00 | 1.95E-05 | 0.0005639 |
| AC092964.1 | 11.90846622 | 5.716793846 | -1.06E+00 | 6.11E-07 | 9.26E-05 |
| AL023775.1 | 2.500228378 | 0.662061538 | -1.92E+00 | 4.42E-05 | 0.0008804 |
| NUP62CL | 0.542331081 | 2.284703077 | 2.07E+00 | 0.0050571 | 0.0198438 |
| TMEM212-AS1 | 1.841572973 | 0.217158462 | -3.08E+00 | 0.0093361 | 0.0305748 |
| VSIR | 150.3791446 | 315.2637415 | 1.07E+00 | 6.53E-07 | 9.35E-05 |
| CLDN10-AS1 | 4.828816216 | 0.775995385 | -2.64E+00 | 0.0068113 | 0.0244202 |
| AC109446.1 | 1.48005 | 0.649855385 | -1.19E+00 | 0.0137548 | 0.0402475 |
| RTN1 | 0.894586486 | 6.777666154 | 2.92E+00 | 4.11E-06 | 0.0002454 |
| C1QA | 21.66890405 | 102.8727231 | 2.25E+00 | 0.0008341 | 0.0056424 |
| UGT2B28 | 4.929751351 | 2.220266154 | -1.15E+00 | 0.0090575 | 0.0299063 |
| SLC11A1 | 23.30370811 | 49.15754769 | 1.08E+00 | 3.42E-05 | 0.000767 |
| NKD2 | 3.213416216 | 1.267861538 | -1.34E+00 | 0.0118782 | 0.0361669 |
| PCBP3-AS1 | 1.923466216 | 0.453104615 | -2.09E+00 | 0.0021017 | 0.0107482 |
| RNU2-64P | 1.732377027 | 0.337478462 | -2.36E+00 | 7.09E-05 | 0.0011805 |
| VNN3 | 1.722304054 | 4.972581538 | 1.53E+00 | 7.21E-06 | 0.0003238 |
| GZMB | 7.858939189 | 18.24369077 | 1.21E+00 | 4.64E-08 | 2.87E-05 |
| CPA3 | 490.3405676 | 155.4779154 | -1.66E+00 | 0.000325 | 0.0030472 |
| AC000065.1 | 48.38394459 | 23.75583846 | -1.03E+00 | 5.61E-08 | 3.08E-05 |
| TMEM63C | 1.037594595 | 2.473478462 | 1.25E+00 | 0.0015679 | 0.0087528 |
| AC023034.1 | 2.170652703 | 0.448461538 | -2.28E+00 | 0.0100257 | 0.0321267 |
| TGFA | 4.517217568 | 2.225029231 | -1.02E+00 | 0.0079748 | 0.027322 |
| NPL | 9.527636486 | 19.48016462 | 1.03E+00 | 0.0005084 | 0.004122 |
| RNU4-50P | 3.180552703 | 1.318301538 | -1.27E+00 | 0.0064573 | 0.0235164 |
| AC092979.2 | 3.491186486 | 1.072861538 | -1.70E+00 | 8.20E-05 | 0.0012903 |
| BASP1 | 34.88512162 | 77.59836 | 1.15E+00 | 0.0001299 | 0.0017266 |
| LINC01503 | 1.246590541 | 2.716149231 | 1.12E+00 | 1.24E-05 | 0.0004275 |
| RGS9BP | 1.78927027 | 0.720467692 | -1.31E+00 | 0.0001367 | 0.0017828 |
| TMPRSS11D | 1.733727027 | 0.784032308 | -1.14E+00 | 2.21E-05 | 0.0006047 |
| AL591684.2 | 2.088156757 | 0.657713846 | -1.67E+00 | 0.0120124 | 0.0364405 |
| IGHJ2 | 40.07390811 | 80.70008462 | 1.01E+00 | 0.0140154 | 0.0408522 |
| HMOX1 | 19.62032838 | 54.32370615 | 1.47E+00 | 4.33E-06 | 0.0002509 |
| FCGR1B | 2.165172973 | 4.461458462 | 1.04E+00 | 0.0010937 | 0.0068069 |
| CXCL10 | 1.731401351 | 5.040853846 | 1.54E+00 | 6.96E-05 | 0.0011666 |
| INHBA | 1.303354054 | 3.014376923 | 1.21E+00 | 0.0046484 | 0.0186571 |
| LDOC1 | 0.737851351 | 2.015398462 | 1.45E+00 | 7.58E-06 | 0.0003349 |
| LGALS2 | 20.43398649 | 42.96511692 | 1.07E+00 | 0.0006468 | 0.0048139 |
| CXCL1 | 3.152318919 | 6.360530769 | 1.01E+00 | 0.0037052 | 0.0158905 |
| CD36 | 46.65041757 | 97.99073385 | 1.07E+00 | 0.0004773 | 0.0039701 |
| SETBP1 | 4.090251351 | 10.27676308 | 1.33E+00 | 0.0089742 | 0.0297273 |
| AQP9 | 9.453924324 | 27.19529385 | 1.52E+00 | 0.0007269 | 0.0051562 |
| RNU6-71P | 1.888648649 | 0.802315385 | -1.24E+00 | 0.0008401 | 0.0056797 |
| MTRF1LP2 | 1.595763514 | 0.606164615 | -1.40E+00 | 0.0008051 | 0.0055314 |
| AC023051.2 | 9.229347297 | 4.25898 | -1.12E+00 | 7.71E-05 | 0.0012374 |
| BATF2 | 0.852932432 | 2.292175385 | 1.43E+00 | 0.0002586 | 0.0026199 |
| NUDT19P5 | 1.455966216 | 3.561063077 | 1.29E+00 | 1.38E-05 | 0.0004574 |
| SLC38A4 | 2.843663514 | 1.37006 | -1.05E+00 | 0.0024895 | 0.0120936 |
| AC215522.2 | 1.876063514 | 5.573655385 | 1.57E+00 | 0.000212 | 0.0023479 |
| SSTR2 | 1.722410811 | 0.511841538 | -1.75E+00 | 6.67E-07 | 9.44E-05 |
| CD300C | 24.9410527 | 52.73506462 | 1.08E+00 | 4.93E-05 | 0.0009464 |
| DPY19L4P1 | 6.43352973 | 2.30596 | -1.48E+00 | 0.0031698 | 0.0142746 |
| TBX1 | 5.388552703 | 2.289087692 | -1.24E+00 | 0.0007848 | 0.0054179 |
| RTN2 | 3.66147027 | 7.614256923 | 1.06E+00 | 0.0002672 | 0.0026749 |
| MYO7A | 2.010424324 | 5.063430769 | 1.33E+00 | 3.99E-06 | 0.0002407 |
| CYP4F11 | 2.186997297 | 0.780609231 | -1.49E+00 | 0.0032118 | 0.0144074 |
| AC004381.1 | 19.63971081 | 8.305936923 | -1.24E+00 | 3.62E-05 | 0.0007855 |
| AP002518.2 | 7.658513514 | 3.37676 | -1.18E+00 | 9.77E-06 | 0.0003814 |
| AL139156.1 | 1.453859459 | 0.724976923 | -1.00E+00 | 0.0008332 | 0.0056424 |
| SECTM1 | 9.331777027 | 27.39123231 | 1.55E+00 | 6.86E-06 | 0.0003163 |
| GPR12 | 2.048877027 | 0.860653846 | -1.25E+00 | 4.82E-05 | 0.0009342 |
| LRRC32 | 0.776959459 | 2.152615385 | 1.47E+00 | 0.0064732 | 0.023555 |
| AJ011931.2 | 1.724628378 | 0.455470769 | -1.92E+00 | 0.0058292 | 0.0218662 |
| DOK2 | 36.07109459 | 75.06419077 | 1.06E+00 | 4.12E-07 | 7.35E-05 |
| EPOR | 10.92350811 | 23.31202462 | 1.09E+00 | 0.0001112 | 0.0015483 |
| HSPA6 | 2.690931081 | 6.875250769 | 1.35E+00 | 3.01E-05 | 0.0007191 |
| KCNH3 | 1.977963514 | 4.193272308 | 1.08E+00 | 6.01E-05 | 0.0010771 |
| LOXL4 | 10.98435676 | 3.973238462 | -1.47E+00 | 0.0019313 | 0.0101279 |
| DBET | 5.078733784 | 2.478850769 | -1.03E+00 | 0.0010458 | 0.0065835 |
| BAG3 | 2.904931081 | 8.966710769 | 1.63E+00 | 2.01E-07 | 6.00E-05 |
| TMEM121 | 2.929182432 | 8.724723077 | 1.57E+00 | 0.0006133 | 0.0046558 |
| AC096631.2 | 1.537662162 | 0.75682 | -1.02E+00 | 0.0006327 | 0.0047371 |
| TSPYL5 | 1.680235135 | 4.903749231 | 1.55E+00 | 0.001512 | 0.0085107 |
| AL031846.1 | 0.641452703 | 1.425386154 | 1.15E+00 | 4.78E-05 | 0.0009307 |
| CHRNA6 | 3.500885135 | 0.798178462 | -2.13E+00 | 0.0039361 | 0.0166367 |
| CALHM6 | 2.786535135 | 5.742606154 | 1.04E+00 | 0.0054396 | 0.0208465 |
| KIR3DL2 | 0.682602703 | 1.442270769 | 1.08E+00 | 6.69E-05 | 0.0011363 |
| HSPA7 | 7.625944595 | 28.13159692 | 1.88E+00 | 0.0005246 | 0.0042142 |
| MOK | 5.385325676 | 2.147613846 | -1.33E+00 | 0.0001489 | 0.0018793 |
| GPAT2 | 0.628691892 | 2.176824615 | 1.79E+00 | 0.0058412 | 0.021904 |
| FGR | 125.5489581 | 256.1294462 | 1.03E+00 | 1.26E-05 | 0.0004325 |
| CD14 | 73.53677027 | 209.5177462 | 1.51E+00 | 1.26E-05 | 0.0004325 |
| TYMP | 35.5710027 | 94.31892615 | 1.41E+00 | 5.40E-06 | 0.00028 |
| AL023775.2 | 2.367852703 | 0.669076923 | -1.82E+00 | 0.0004515 | 0.0038441 |
| FFAR2 | 2.487916216 | 7.11778 | 1.52E+00 | 7.58E-06 | 0.0003349 |
| LTB | 18.45165135 | 38.53970615 | 1.06E+00 | 9.05E-06 | 0.0003679 |
| AP001434.1 | 0.396333784 | 1.8079 | 2.19E+00 | 0.0059702 | 0.0222322 |
| FAM157C | 1.314602703 | 3.036552308 | 1.21E+00 | 0.0009486 | 0.0061647 |
| AL359091.2 | 1.836371622 | 0.790590769 | -1.22E+00 | 0.0005763 | 0.0044727 |
| SEPTIN7P8 | 0.676317568 | 1.502950769 | 1.15E+00 | 0.0077662 | 0.0268384 |
| AFF2 | 22.73379459 | 6.792538462 | -1.74E+00 | 0.0013355 | 0.0078487 |
| AC000065.2 | 39.39653108 | 19.21629385 | -1.04E+00 | 4.80E-07 | 8.00E-05 |
| S100A6 | 184.2286203 | 373.3509185 | 1.02E+00 | 2.01E-07 | 6.00E-05 |
| PRRT4 | 25.45860135 | 8.906809231 | -1.52E+00 | 2.19E-05 | 0.0006014 |
| AC023421.2 | 4.866135135 | 2.182361538 | -1.16E+00 | 5.74E-06 | 0.000288 |
| SPOCK3 | 0.337204054 | 2.359833846 | 2.81E+00 | 0.0003317 | 0.0030867 |
| AC005392.2 | 5.138101351 | 10.97065077 | 1.09E+00 | 0.0117741 | 0.0359501 |
| TUBB1 | 6.569609459 | 13.82896615 | 1.07E+00 | 0.0066831 | 0.0240546 |
| CCL3 | 1.291608108 | 2.964632308 | 1.20E+00 | 0.0001427 | 0.0018391 |
| AC244035.1 | 2.861058108 | 1.400036923 | -1.03E+00 | 0.0070688 | 0.0250776 |
| LINC02593 | 12.31152432 | 5.671989231 | -1.12E+00 | 0.0036069 | 0.0156073 |
| BCL2A1 | 26.98235135 | 68.84896769 | 1.35E+00 | 3.75E-06 | 0.0002326 |
| AC092979.1 | 2.071258108 | 0.623616923 | -1.73E+00 | 9.20E-06 | 0.0003679 |
| AP000763.2 | 107.3415473 | 50.48527077 | -1.09E+00 | 0.0023872 | 0.0117415 |
| SIGLEC7 | 5.294132432 | 12.43504154 | 1.23E+00 | 2.69E-05 | 0.0006747 |
| AL133492.1 | 2.294458108 | 0.537176923 | -2.09E+00 | 0.0005944 | 0.0045656 |
| FCGR3B | 2.57047973 | 10.54136615 | 2.04E+00 | 0.0125214 | 0.0374823 |
| FXYD6 | 1.237490541 | 3.940023077 | 1.67E+00 | 4.67E-05 | 0.0009151 |
| LILRB2 | 38.58097432 | 97.29066154 | 1.33E+00 | 6.60E-06 | 0.0003084 |
| SLC8A1-AS1 | 1.100754054 | 2.5662 | 1.22E+00 | 4.27E-05 | 0.0008604 |
| LINC01262 | 2.170817568 | 0.845609231 | -1.36E+00 | 0.0023946 | 0.0117752 |
| IGLL1 | 180.3294459 | 88.02348923 | -1.03E+00 | 0.0119428 | 0.0362409 |
| GXYLT2 | 4.796281081 | 0.771470769 | -2.64E+00 | 0.003655 | 0.0157437 |
| SLC9A9 | 4.203795946 | 9.65376 | 1.20E+00 | 5.49E-05 | 0.0010163 |
| MAMDC2-AS1 | 4.688693243 | 1.820549231 | -1.36E+00 | 0.0014264 | 0.0081678 |
| BEX2 | 4.674641892 | 11.27734308 | 1.27E+00 | 3.00E-06 | 0.0002121 |
| RNU6-972P | 4.91292973 | 2.296373846 | -1.10E+00 | 4.71E-06 | 0.0002595 |
| AC023051.1 | 12.52211757 | 5.832549231 | -1.10E+00 | 1.77E-08 | 2.09E-05 |
