# Supplementary material for: Cuproptosis-related lncRNA signature for prognostic prediction in patients with acute myeloid leukemia
Source: BMC Bioinformatics. 2023 Feb 3;24:37. doi: 10.1186/s12859-023-05148-9 (PMC9896718; doi:10.1186/s12859-023-05148-9)
Supplement: Supplementary file 5 — Additional file 5. Data analysis results. [file 12859_2023_5148_MOESM5_ESM.docx]

**Supplementary Table S5. Data analysis results**

| Id | futime | fustat | Age | Gender | Grade | Stage | T | M | N |
| --- | --- | --- | --- | --- | --- | --- | --- | --- | --- |
| TCGA-AB-2884 | 731 | 1 | 44 | FEMALE | unknow | unknow | unknow | unknow | unknow |
| TCGA-AB-2969 | 1127 | 0 | 55 | MALE | unknow | unknow | unknow | unknow | unknow |
| TCGA-AB-2972 | 30 | 1 | 82 | FEMALE | unknow | unknow | unknow | unknow | unknow |
| TCGA-AB-2888 | 334 | 0 | 57 | MALE | unknow | unknow | unknow | unknow | unknow |
| TCGA-AB-2909 | 30 | 0 | 22 | MALE | unknow | unknow | unknow | unknow | unknow |
| TCGA-AB-2980 | 699 | 0 | 50 | MALE | unknow | unknow | unknow | unknow | unknow |
| TCGA-AB-2919 | 61 | 0 | 54 | FEMALE | unknow | unknow | unknow | unknow | unknow |
| TCGA-AB-2950 | 306 | 0 | 34 | FEMALE | unknow | unknow | unknow | unknow | unknow |
| TCGA-AB-2936 | 59 | 0 | 61 | FEMALE | unknow | unknow | unknow | unknow | unknow |
| TCGA-AB-2974 | 92 | 1 | 67 | FEMALE | unknow | unknow | unknow | unknow | unknow |
| TCGA-AB-3012 | 1887 | 0 | 53 | MALE | unknow | unknow | unknow | unknow | unknow |
| TCGA-AB-2804 | 2557 | 0 | 30 | MALE | unknow | unknow | unknow | unknow | unknow |
| TCGA-AB-2985 | 273 | 1 | 81 | FEMALE | unknow | unknow | unknow | unknow | unknow |
| TCGA-AB-2966 | 854 | 1 | 57 | FEMALE | unknow | unknow | unknow | unknow | unknow |
| TCGA-AB-2824 | 30 | 1 | 45 | MALE | unknow | unknow | unknow | unknow | unknow |
| TCGA-AB-2873 | 273 | 0 | 51 | FEMALE | unknow | unknow | unknow | unknow | unknow |
| TCGA-AB-2875 | 212 | 0 | 43 | MALE | unknow | unknow | unknow | unknow | unknow |
| TCGA-AB-2918 | | 1 | 47 | FEMALE | unknow | unknow | unknow | unknow | unknow |
| TCGA-AB-2826 | 731 | 1 | 64 | FEMALE | unknow | unknow | unknow | unknow | unknow |
| TCGA-AB-2934 | 28 | 0 | 65 | MALE | unknow | unknow | unknow | unknow | unknow |
| TCGA-AB-2876 | 1219 | 0 | 45 | FEMALE | unknow | unknow | unknow | unknow | unknow |
| TCGA-AB-2860 | 427 | 1 | 60 | FEMALE | unknow | unknow | unknow | unknow | unknow |
| TCGA-AB-2850 | 61 | 1 | 72 | FEMALE | unknow | unknow | unknow | unknow | unknow |
| TCGA-AB-2883 | 730 | 0 | 60 | MALE | unknow | unknow | unknow | unknow | unknow |
| TCGA-AB-2940 | | 1 | 35 | MALE | unknow | unknow | unknow | unknow | unknow |
| TCGA-AB-2856 | 150 | 1 | 63 | MALE | unknow | unknow | unknow | unknow | unknow |
| TCGA-AB-2914 | 792 | 0 | 22 | FEMALE | unknow | unknow | unknow | unknow | unknow |
| TCGA-AB-2885 | 214 | 1 | 71 | MALE | unknow | unknow | unknow | unknow | unknow |
| TCGA-AB-2818 | 303 | 1 | 62 | FEMALE | unknow | unknow | unknow | unknow | unknow |
| TCGA-AB-2811 | 243 | 1 | 81 | MALE | unknow | unknow | unknow | unknow | unknow |
| TCGA-AB-2848 | 455 | 1 | 62 | MALE | unknow | unknow | unknow | unknow | unknow |
| TCGA-AB-2907 | | 1 | 69 | MALE | unknow | unknow | unknow | unknow | unknow |
| TCGA-AB-2882 | 365 | 1 | 73 | FEMALE | unknow | unknow | unknow | unknow | unknow |
| TCGA-AB-2948 | 580 | 1 | 67 | MALE | unknow | unknow | unknow | unknow | unknow |
| TCGA-AB-2965 | 335 | 1 | 60 | MALE | unknow | unknow | unknow | unknow | unknow |
| TCGA-AB-2978 | 699 | 0 | 61 | FEMALE | unknow | unknow | unknow | unknow | unknow |
| TCGA-AB-2991 | 1826 | 0 | 40 | FEMALE | unknow | unknow | unknow | unknow | unknow |
| TCGA-AB-3007 | 1581 | 0 | 35 | MALE | unknow | unknow | unknow | unknow | unknow |
| TCGA-AB-2986 | 212 | 1 | 31 | FEMALE | unknow | unknow | unknow | unknow | unknow |
| TCGA-AB-2913 | 1216 | 0 | 61 | MALE | unknow | unknow | unknow | unknow | unknow |
| TCGA-AB-2944 | | 1 | 48 | MALE | unknow | unknow | unknow | unknow | unknow |
| TCGA-AB-2815 | 822 | 1 | 49 | MALE | unknow | unknow | unknow | unknow | unknow |
| TCGA-AB-2812 | 366 | 1 | 25 | FEMALE | unknow | unknow | unknow | unknow | unknow |
| TCGA-AB-2834 | 245 | 1 | 33 | MALE | unknow | unknow | unknow | unknow | unknow |
| TCGA-AB-2996 | 1581 | 0 | 74 | MALE | unknow | unknow | unknow | unknow | unknow |
| TCGA-AB-2990 | 457 | 0 | 51 | MALE | unknow | unknow | unknow | unknow | unknow |
| TCGA-AB-2891 | | 1 | 42 | MALE | unknow | unknow | unknow | unknow | unknow |
| TCGA-AB-2922 | 0 | 1 | 83 | MALE | unknow | unknow | unknow | unknow | unknow |
| TCGA-AB-3009 | 577 | 1 | 23 | MALE | unknow | unknow | unknow | unknow | unknow |
| TCGA-AB-2949 | 699 | 0 | 58 | MALE | unknow | unknow | unknow | unknow | unknow |
| TCGA-AB-2840 | 28 | 1 | 74 | MALE | unknow | unknow | unknow | unknow | unknow |
| TCGA-AB-2845 | 304 | 1 | 37 | FEMALE | unknow | unknow | unknow | unknow | unknow |
| TCGA-AB-2964 | 1277 | 0 | 58 | FEMALE | unknow | unknow | unknow | unknow | unknow |
| TCGA-AB-2938 | 304 | 1 | 76 | MALE | unknow | unknow | unknow | unknow | unknow |
| TCGA-AB-2889 | 304 | 0 | 55 | MALE | unknow | unknow | unknow | unknow | unknow |
| TCGA-AB-2968 | 458 | 1 | 79 | MALE | unknow | unknow | unknow | unknow | unknow |
| TCGA-AB-2942 | 641 | 0 | 67 | FEMALE | unknow | unknow | unknow | unknow | unknow |
| TCGA-AB-2983 | 335 | 1 | 45 | MALE | unknow | unknow | unknow | unknow | unknow |
| TCGA-AB-2900 | 184 | 1 | 70 | MALE | unknow | unknow | unknow | unknow | unknow |
| TCGA-AB-2956 | 183 | 1 | 61 | MALE | unknow | unknow | unknow | unknow | unknow |
| TCGA-AB-2929 | 123 | 1 | 71 | FEMALE | unknow | unknow | unknow | unknow | unknow |
| TCGA-AB-2844 | 122 | 1 | 63 | MALE | unknow | unknow | unknow | unknow | unknow |
| TCGA-AB-2946 | | 0 | 41 | MALE | unknow | unknow | unknow | unknow | unknow |
| TCGA-AB-2880 | | 1 | 24 | MALE | unknow | unknow | unknow | unknow | unknow |
| TCGA-AB-2835 | 1673 | 0 | 48 | MALE | unknow | unknow | unknow | unknow | unknow |
| TCGA-AB-2823 | 0 | 1 | 61 | FEMALE | unknow | unknow | unknow | unknow | unknow |
| TCGA-AB-2809 | 62 | 1 | 64 | FEMALE | unknow | unknow | unknow | unknow | unknow |
| TCGA-AB-2982 | 150 | 0 | 29 | FEMALE | unknow | unknow | unknow | unknow | unknow |
| TCGA-AB-2854 | 366 | 1 | 51 | FEMALE | unknow | unknow | unknow | unknow | unknow |
| TCGA-AB-2863 | 31 | 1 | 63 | MALE | unknow | unknow | unknow | unknow | unknow |
| TCGA-AB-2838 | 365 | 1 | 67 | MALE | unknow | unknow | unknow | unknow | unknow |
| TCGA-AB-2995 | 1551 | 0 | 63 | MALE | unknow | unknow | unknow | unknow | unknow |
| TCGA-AB-2895 | 153 | 1 | 41 | FEMALE | unknow | unknow | unknow | unknow | unknow |
| TCGA-AB-2807 | 181 | 1 | 68 | FEMALE | unknow | unknow | unknow | unknow | unknow |
| TCGA-AB-2931 | 0 | 1 | 75 | MALE | unknow | unknow | unknow | unknow | unknow |
| TCGA-AB-2920 | 366 | 1 | 44 | MALE | unknow | unknow | unknow | unknow | unknow |
| TCGA-AB-2906 | 577 | 0 | 59 | MALE | unknow | unknow | unknow | unknow | unknow |
| TCGA-AB-2943 | | 1 | 70 | FEMALE | unknow | unknow | unknow | unknow | unknow |
| TCGA-AB-2941 | 0 | 1 | 73 | MALE | unknow | unknow | unknow | unknow | unknow |
| TCGA-AB-3005 | 2100 | 0 | 45 | MALE | unknow | unknow | unknow | unknow | unknow |
| TCGA-AB-2915 | 0 | 1 | 73 | FEMALE | unknow | unknow | unknow | unknow | unknow |
| TCGA-AB-2988 | 30 | 1 | 67 | FEMALE | unknow | unknow | unknow | unknow | unknow |
| TCGA-AB-2868 | 151 | 1 | 77 | MALE | unknow | unknow | unknow | unknow | unknow |
| TCGA-AB-2971 | 792 | 1 | 76 | FEMALE | unknow | unknow | unknow | unknow | unknow |
| TCGA-AB-2955 | 489 | 1 | 56 | FEMALE | unknow | unknow | unknow | unknow | unknow |
| TCGA-AB-2803 | 792 | 1 | 61 | FEMALE | unknow | unknow | unknow | unknow | unknow |
| TCGA-AB-2939 | 455 | 0 | 72 | MALE | unknow | unknow | unknow | unknow | unknow |
| TCGA-AB-2894 | 181 | 1 | 50 | FEMALE | unknow | unknow | unknow | unknow | unknow |
| TCGA-AB-2987 | 184 | 1 | 75 | FEMALE | unknow | unknow | unknow | unknow | unknow |
| TCGA-AB-2879 | 245 | 0 | 68 | FEMALE | unknow | unknow | unknow | unknow | unknow |
| TCGA-AB-3011 | 1885 | 0 | 21 | FEMALE | unknow | unknow | unknow | unknow | unknow |
| TCGA-AB-2821 | 822 | 1 | 64 | MALE | unknow | unknow | unknow | unknow | unknow |
| TCGA-AB-2849 | 2220 | 0 | 39 | MALE | unknow | unknow | unknow | unknow | unknow |
| TCGA-AB-2975 | | 1 | 54 | MALE | unknow | unknow | unknow | unknow | unknow |
| TCGA-AB-2917 | 1216 | 0 | 41 | FEMALE | unknow | unknow | unknow | unknow | unknow |
| TCGA-AB-2967 | 1035 | 0 | 58 | MALE | unknow | unknow | unknow | unknow | unknow |
| TCGA-AB-2932 | 0 | 0 | 62 | MALE | unknow | unknow | unknow | unknow | unknow |
| TCGA-AB-2841 | 1401 | 1 | 51 | FEMALE | unknow | unknow | unknow | unknow | unknow |
| TCGA-AB-2977 | 31 | 1 | 71 | FEMALE | unknow | unknow | unknow | unknow | unknow |
| TCGA-AB-2831 | 640 | 1 | 59 | MALE | unknow | unknow | unknow | unknow | unknow |
| TCGA-AB-2887 | | 1 | 60 | FEMALE | unknow | unknow | unknow | unknow | unknow |
| TCGA-AB-2886 | 181 | 0 | 26 | MALE | unknow | unknow | unknow | unknow | unknow |
| TCGA-AB-2893 | 212 | 1 | 45 | MALE | unknow | unknow | unknow | unknow | unknow |
| TCGA-AB-2820 | 0 | 1 | 76 | MALE | unknow | unknow | unknow | unknow | unknow |
| TCGA-AB-2908 | 31 | 1 | 81 | MALE | unknow | unknow | unknow | unknow | unknow |
| TCGA-AB-2993 | 761 | 1 | 57 | FEMALE | unknow | unknow | unknow | unknow | unknow |
| TCGA-AB-2903 | 0 | 1 | 76 | FEMALE | unknow | unknow | unknow | unknow | unknow |
| TCGA-AB-2842 | 0 | 1 | 65 | MALE | unknow | unknow | unknow | unknow | unknow |
| TCGA-AB-2935 | 61 | 1 | 66 | MALE | unknow | unknow | unknow | unknow | unknow |
| TCGA-AB-2851 | 242 | 1 | 66 | FEMALE | unknow | unknow | unknow | unknow | unknow |
| TCGA-AB-2945 | 243 | 1 | 65 | FEMALE | unknow | unknow | unknow | unknow | unknow |
| TCGA-AB-2933 | 122 | 1 | 58 | MALE | unknow | unknow | unknow | unknow | unknow |
| TCGA-AB-2858 | 577 | 1 | 75 | FEMALE | unknow | unknow | unknow | unknow | unknow |
| TCGA-AB-2859 | 304 | 1 | 40 | MALE | unknow | unknow | unknow | unknow | unknow |
| TCGA-AB-2997 | | 1 | 25 | FEMALE | unknow | unknow | unknow | unknow | unknow |
| TCGA-AB-2866 | 153 | 1 | 67 | MALE | unknow | unknow | unknow | unknow | unknow |
| TCGA-AB-2979 | 671 | 0 | 30 | FEMALE | unknow | unknow | unknow | unknow | unknow |
| TCGA-AB-2833 | 456 | 1 | 77 | MALE | unknow | unknow | unknow | unknow | unknow |
| TCGA-AB-2959 | 489 | 1 | 71 | MALE | unknow | unknow | unknow | unknow | unknow |
| TCGA-AB-2869 | 243 | 0 | 64 | FEMALE | unknow | unknow | unknow | unknow | unknow |
| TCGA-AB-2816 | 245 | 1 | 57 | FEMALE | unknow | unknow | unknow | unknow | unknow |
| TCGA-AB-2836 | 518 | 1 | 35 | MALE | unknow | unknow | unknow | unknow | unknow |
| TCGA-AB-2808 | 2861 | 0 | 23 | MALE | unknow | unknow | unknow | unknow | unknow |
| TCGA-AB-2899 | 671 | 1 | 76 | FEMALE | unknow | unknow | unknow | unknow | unknow |
| TCGA-AB-2855 | 212 | 1 | 18 | MALE | unknow | unknow | unknow | unknow | unknow |
| TCGA-AB-2802 | 365 | 1 | 50 | MALE | unknow | unknow | unknow | unknow | unknow |
| TCGA-AB-2819 | 2496 | 0 | 52 | FEMALE | unknow | unknow | unknow | unknow | unknow |
| TCGA-AB-3008 | 822 | 1 | 22 | MALE | unknow | unknow | unknow | unknow | unknow |
| TCGA-AB-2970 | 305 | 1 | 34 | FEMALE | unknow | unknow | unknow | unknow | unknow |
| TCGA-AB-2874 | 396 | 0 | 59 | MALE | unknow | unknow | unknow | unknow | unknow |
| TCGA-AB-2892 | 943 | 0 | 42 | FEMALE | unknow | unknow | unknow | unknow | unknow |
| TCGA-AB-2830 | 275 | 1 | 64 | FEMALE | unknow | unknow | unknow | unknow | unknow |
| TCGA-AB-2973 | 609 | 1 | 68 | FEMALE | unknow | unknow | unknow | unknow | unknow |
| TCGA-AB-2981 | 487 | 0 | 35 | FEMALE | unknow | unknow | unknow | unknow | unknow |
| TCGA-AB-2925 | 243 | 1 | 57 | MALE | unknow | unknow | unknow | unknow | unknow |
| TCGA-AB-2829 | 273 | 1 | 50 | MALE | unknow | unknow | unknow | unknow | unknow |
| TCGA-AB-2897 | 243 | 0 | 50 | FEMALE | unknow | unknow | unknow | unknow | unknow |
| TCGA-AB-2898 | 393 | 0 | 69 | FEMALE | unknow | unknow | unknow | unknow | unknow |
| TCGA-AB-2872 | 638 | 0 | 42 | MALE | unknow | unknow | unknow | unknow | unknow |
| TCGA-AB-2916 | 882 | 0 | 48 | FEMALE | unknow | unknow | unknow | unknow | unknow |
| TCGA-AB-2896 | 214 | 1 | 21 | FEMALE | unknow | unknow | unknow | unknow | unknow |
| TCGA-AB-2847 | 608 | 1 | 53 | MALE | unknow | unknow | unknow | unknow | unknow |
| TCGA-AB-3001 | 1581 | 0 | 31 | FEMALE | unknow | unknow | unknow | unknow | unknow |
| TCGA-AB-2839 | 486 | 1 | 51 | FEMALE | unknow | unknow | unknow | unknow | unknow |
| TCGA-AB-2994 | 1798 | 0 | 25 | MALE | unknow | unknow | unknow | unknow | unknow |
| TCGA-AB-2813 | 31 | 1 | 78 | MALE | unknow | unknow | unknow | unknow | unknow |
| TCGA-AB-2998 | 31 | 1 | 68 | FEMALE | unknow | unknow | unknow | unknow | unknow |
| TCGA-AB-2814 | 792 | 1 | 39 | FEMALE | unknow | unknow | unknow | unknow | unknow |
| TCGA-AB-2926 | 457 | 1 | 57 | FEMALE | unknow | unknow | unknow | unknow | unknow |
| TCGA-AB-2976 | 915 | 1 | 53 | MALE | unknow | unknow | unknow | unknow | unknow |
| TCGA-AB-2904 | 517 | 1 | 65 | MALE | unknow | unknow | unknow | unknow | unknow |
| TCGA-AB-2805 | 577 | 1 | 77 | MALE | unknow | unknow | unknow | unknow | unknow |
| TCGA-AB-2992 | 1706 | 1 | 32 | FEMALE | unknow | unknow | unknow | unknow | unknow |
| TCGA-AB-2954 | 1492 | 0 | 55 | FEMALE | unknow | unknow | unknow | unknow | unknow |
| TCGA-AB-2890 | 0 | 1 | 69 | MALE | unknow | unknow | unknow | unknow | unknow |
| TCGA-AB-2827 | 273 | 1 | 33 | MALE | unknow | unknow | unknow | unknow | unknow |
| TCGA-AB-2989 | 365 | 1 | 29 | MALE | unknow | unknow | unknow | unknow | unknow |
| TCGA-AB-2877 | 638 | 0 | 60 | FEMALE | unknow | unknow | unknow | unknow | unknow |
| TCGA-AB-2881 | 393 | 0 | 48 | FEMALE | unknow | unknow | unknow | unknow | unknow |
| TCGA-AB-2999 | 1735 | 0 | 62 | MALE | unknow | unknow | unknow | unknow | unknow |
| TCGA-AB-2825 | 212 | 1 | 31 | FEMALE | unknow | unknow | unknow | unknow | unknow |
| TCGA-AB-2905 | 1611 | 0 | 48 | MALE | unknow | unknow | unknow | unknow | unknow |
| TCGA-AB-2865 | 61 | 1 | 75 | MALE | unknow | unknow | unknow | unknow | unknow |
| TCGA-AB-2927 | 90 | 1 | 88 | FEMALE | unknow | unknow | unknow | unknow | unknow |
| TCGA-AB-2862 | 1430 | 0 | 33 | FEMALE | unknow | unknow | unknow | unknow | unknow |
| TCGA-AB-2924 | 90 | 0 | 59 | MALE | unknow | unknow | unknow | unknow | unknow |
| TCGA-AB-2947 | 31 | 0 | 52 | MALE | unknow | unknow | unknow | unknow | unknow |
| TCGA-AB-2871 | 153 | 0 | 51 | MALE | unknow | unknow | unknow | unknow | unknow |
| TCGA-AB-2952 | 31 | 1 | 60 | FEMALE | unknow | unknow | unknow | unknow | unknow |
| TCGA-AB-2930 | | 1 | 63 | FEMALE | unknow | unknow | unknow | unknow | unknow |
| TCGA-AB-2832 | 365 | 1 | 60 | FEMALE | unknow | unknow | unknow | unknow | unknow |
| TCGA-AB-2878 | 365 | 1 | 47 | FEMALE | unknow | unknow | unknow | unknow | unknow |
| TCGA-AB-2837 | 0 | 1 | 66 | FEMALE | unknow | unknow | unknow | unknow | unknow |
| TCGA-AB-2870 | 153 | 1 | 76 | MALE | unknow | unknow | unknow | unknow | unknow |
| TCGA-AB-2857 | 306 | 1 | 54 | MALE | unknow | unknow | unknow | unknow | unknow |
| TCGA-AB-2901 | 59 | 0 | 27 | MALE | unknow | unknow | unknow | unknow | unknow |
| TCGA-AB-2937 | 215 | 1 | 36 | FEMALE | unknow | unknow | unknow | unknow | unknow |
| TCGA-AB-2911 | 1186 | 0 | 51 | FEMALE | unknow | unknow | unknow | unknow | unknow |
| TCGA-AB-2910 | 0 | 1 | 61 | FEMALE | unknow | unknow | unknow | unknow | unknow |
| TCGA-AB-3002 | 1431 | 1 | 68 | MALE | unknow | unknow | unknow | unknow | unknow |
| TCGA-AB-2923 | 31 | 1 | 78 | MALE | unknow | unknow | unknow | unknow | unknow |
| TCGA-AB-2864 | 577 | 1 | 54 | FEMALE | unknow | unknow | unknow | unknow | unknow |
| TCGA-AB-2846 | 1402 | 1 | 57 | FEMALE | unknow | unknow | unknow | unknow | unknow |
| TCGA-AB-2921 | | 1 | 56 | FEMALE | unknow | unknow | unknow | unknow | unknow |
| TCGA-AB-2984 | 1157 | 0 | 38 | MALE | unknow | unknow | unknow | unknow | unknow |
| TCGA-AB-2822 | 973 | 1 | 65 | MALE | unknow | unknow | unknow | unknow | unknow |
| TCGA-AB-3006 | 485 | 1 | 61 | MALE | unknow | unknow | unknow | unknow | unknow |
| TCGA-AB-2843 | 215 | 1 | 77 | MALE | unknow | unknow | unknow | unknow | unknow |
| TCGA-AB-2867 | 184 | 1 | 66 | FEMALE | unknow | unknow | unknow | unknow | unknow |
| TCGA-AB-2806 | 945 | 1 | 46 | MALE | unknow | unknow | unknow | unknow | unknow |
| TCGA-AB-3000 | 1249 | 0 | 25 | MALE | unknow | unknow | unknow | unknow | unknow |
| TCGA-AB-2828 | 2284 | 0 | 55 | MALE | unknow | unknow | unknow | unknow | unknow |
| TCGA-AB-2817 | 273 | 1 | 64 | MALE | unknow | unknow | unknow | unknow | unknow |
| TCGA-AB-2928 | | 1 | 43 | FEMALE | unknow | unknow | unknow | unknow | unknow |
| TCGA-AB-2853 | 89 | 1 | 51 | MALE | unknow | unknow | unknow | unknow | unknow |
| TCGA-AB-2861 | 0 | 1 | 76 | MALE | unknow | unknow | unknow | unknow | unknow |
| TCGA-AB-2810 | 31 | 1 | 76 | FEMALE | unknow | unknow | unknow | unknow | unknow |
| TCGA-AB-2957 | 609 | 0 | 31 | MALE | unknow | unknow | unknow | unknow | unknow |
| TCGA-AB-2963 | 1642 | 1 | 56 | MALE | unknow | unknow | unknow | unknow | unknow |
| TCGA-AB-2912 | 274 | 1 | 63 | MALE | unknow | unknow | unknow | unknow | unknow |
